# Synthesis, Evaluation of Cytotoxicity and Molecular Docking Studies of the 7-Acetamido Substituted 2-Aryl-5-bromo-3-trifluoroacetylindoles as Potential Inhibitors of Tubulin Polymerization

**DOI:** 10.3390/ph11020059

**Published:** 2018-06-11

**Authors:** Malose J. Mphahlele, Nishal Parbhoo

**Affiliations:** 1 Department of Chemistry, College of Science, Engineering and Technology, University of South Africa, Private Bag X06, Florida 1710, South Africa; 2 Department of Life & Consumer Sciences, College of Agriculture and Environmental Sciences, University of South Africa, Private Bag X06, Florida 1710, South Africa; parbhn1@unisa.ac.za

**Keywords:** 7-acetamido-2-aryl-5-bromoindoles, trifluoroacetylation, cytotoxicity, apoptosis, tubulin polymerization, molecular docking

## Abstract

The 3-trifluoroacetyl–substituted 7-acetamido-2-aryl-5-bromoindoles **5a**–**h** were prepared and evaluated for potential antigrowth effect in vitro against human lung cancer (A549) and cervical cancer (HeLa) cells and for the potential to inhibit tubulin polymerization. The corresponding intermediates, namely, the 3-unsubstituted 7-acetyl-2-aryl-5-bromoindole **2a**–**d** and 7-acetamido-2-aryl-5-bromoindole **4a**–**d** were included in the assays in order to correlate both structural variations and cytotoxicity. No cytotoxicity was observed for compounds **2a**–**d** and their 3-trifluoroacetyl–substituted derivatives **5a**–**d** against both cell lines. The 7-acetamido derivatives **4**–**d** exhibited modest cytotoxicity against both cell lines. All of the 3-trifluoroacetyl–substituted 7-acetamido-2-aryl-5-bromoindoles **5e**–**h** were found to be more active against both cell lines when compared to the chemotherapeutic drug, Melphalan. The most active compound, **5g**, induced programmed cell death (apoptosis) in a caspase-dependent manner for both A549 and HeLa cells. Compounds **5e**–**h** were found to significantly inhibit tubulin polymerization against indole-3-carbinol and colchicine as reference standards. Molecular docking of **5g** into the colchicine-binding site suggests that the compounds bind to tubulin by different type of interactions including pi-alkyl, amide-pi stacked and alkyl interactions as well as hydrogen bonding with the protein residues to elicit anticancer activity.

## 1. Introduction

**Figure 1:**
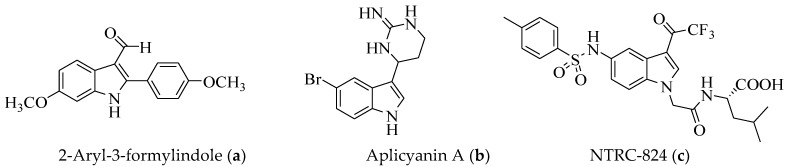
Structures of 3-formyl substituted 2-arylindole (**a**), aplicyanin A (**b**) and NTRC-824 (**c**).

Microtubule targeting agents have an established history of utility in the treatment of cancer and have been instrumental as biological probes to identify the nature of tubulin and the role of tubulin dynamics in mitosis [1]. Colchicine is known to bind to tubulin and block the formation of microtubules while the other anticancer agents stabilize the tubulin structure and, therefore, prevent microtubule disassembly [2]. Nitrogen-containing heterocycles such as indoles with anticancer properties have the potential to inhibit tubulin polymerization by binding to colchicine-binding site [3,4,5]. The methoxy-substituted 2-aryl-3-formylindoles (**a**) shown in Figure 1, for example, have been found to completely block the microtubule assembly at micromolar concentrations, which suggests a correlation between cytotoxicity and the microtubule system [3]. Structure–activity relationship (SAR) studies of indole derivatives revealed that a hydrogen bond donor NH at position 1 is essential for their antiproliferative activity [3]. A nitrogen-containing group on the fused benzo ring of the indole derivatives, on the other hand, was found to lead to increased cytotoxicity against the HeLa and A549 cell lines as well as HIV-1 inhibition activity [6]. Likewise, the presence of a lipophilic bromine atom on the fused benzo ring of an indole framework was found to impart significant anti-tumour activity in both the synthetic [7] and the naturally [8] occurring indole derivatives. Aplicyanin A (**b**) shown in Figure 1, for example, is a 5-bromoindole–based compound previously isolated from ascidian *Aplidium cyaneum* and this compound has been found to exhibit increased antiproliferative activity against MDA-MB-231, A549 and HT-29 cancer cell lines [8]. A hydrogen bond acceptor such as a formyl group at position 3 of the indole framework of compound (**a**), for example, facilitates interaction with biological receptors and therefore enhance anticancer activity [9]. Thomas et al. have previously isolated *N*-[(5-{[(4-methylphenyl)sulfonyl]amino}-3-(trifluoroacetyl)-1*H*-indol-1-yl)acetyl]-*L*-leucine (NTRC-824) shown in Figure 1 as an impurity and found it to be 90-fold more active (IC_50_ = 38 nM) and selective for the neurotesin receptor type 2 (NTS2 *versus* NTS1) than the expected C-3 unsubstituted analogue (IC_50_ = 3322 nM) [10]. The enhanced activity of NTRC-824 due to the presence of a trifluoroacetyl group is presumably due to the increased electron withdrawing effect of the trifluoromethyl group. Literature precedents revealed that the presence of a trifluoromethyl group at the C-3 position of an indole framework results in increased affinity for lipids (lipophilicity) and metabolic stability of the molecule as well as its activity profile more so than the 3-unsubstituted or 3-acetyl analogues [2,11,12,13].

We considered the antiproliferative properties of the 2-arylindoles [14] in combination with the above literature SAR analysis to introduce a trifluoroacetyl group at position-3 of the 7-acetyl-2-aryl-5-bromoindoles and the 7-acetamido-2-aryl-5-bromoindoles. The main aim was to evaluate the effect of incorporating a trifluoroacetyl group on the biological activity of the polysubstituted indole derivatives. The prepared 3-trifluoroacetyl–substituted indole derivatives and the corresponding synthetic intermediates, namely, the 7-acetyl-2-aryl-5-bromoindoles and the 7-acetamido-2-aryl-5-bromoindoles were evaluated for cytotoxicity against the human lung cancer (A549) and cervical cancer (HeLa) cell lines in order to correlate between both structural variations and cytotoxicity. The pro-apoptotic properties of the analogous indole-3-carbinol [15] encouraged us to elucidate their mechanism of cancer cell death and for their ability to inhibit tubulin polymerization.

## 2. Results and Discussion

### 2.1. Chemistry

**Scheme 1:**
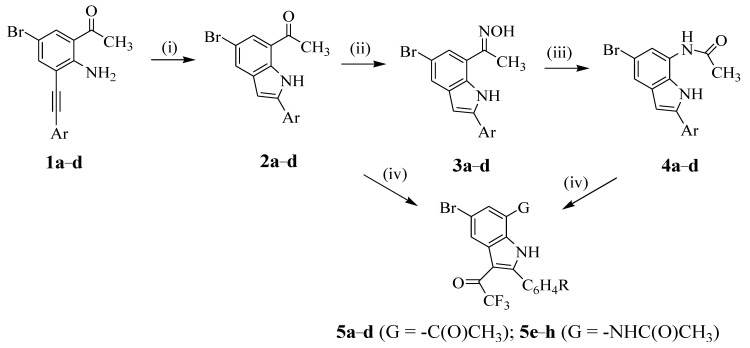
Reaction steps involved in the synthesis of compounds **5a**–**h**. Reagents and conditions: (i) PdCl_2_, CH_3_CN, reflux, 3 h; (ii) NH_2_OH.HCl, pyridine, EtOH, reflux, 5 h; (iii) TFA, CH_3_CN, reflux, 2 h; (iv) (CF_3_CO)_2_O, THF, reflux 5 h.

The title compounds were prepared via a series of steps involving different intermediate products as shown in Scheme 1 and the corresponding yields are listed in Table 1. We first subjected the 3-arylalkynylated 2-amino-5-bromoacetophenone derivatives **1a**–**d** to palladium chloride-mediated endo-dig cyclization to afford the 7-acetyl-2-aryl-5-bromoindoles **2a**–**d** in high yields. The indole derivatives **2a**–**d** were, in turn, reacted with a hydroxylamine hydrochloride- pyridine mixture in ethanol under reflux to afford the oxime derivatives **3a**–**d**. The ^1^H-NMR spectra of the indole derivatives **2a**–**d** revealed the presence of two singlets around δ 6.90 and 11.10 ppm, which correspond to H-3 and NH, respectively. The oxime nature of **3a**–**d**, on the other hand, was confirmed by the additional singlet around δ 11.6 ppm and carbon signal significantly upfield around δ_C=N_ 154 ppm in their ^1^H-NMR and ^13^C-NMR spectra, respectively. The oxime derivatives **3a**–**d** were, in turn, subjected to trifluoroacetic acid (TFA)-mediated Beckmann rearrangement in acetonitrile under reflux for 2 h to afford the corresponding 7-acetamido-2-aryl-5-bromoindoles **4a**–**d** (Scheme 1, Table 1). The NH proton of the amide group resonates as a singlet around δ 9.70 ppm in the proton NMR spectra of these compounds. The methyl protons of these compounds resonate significantly up-field around δ 2.18 ppm compared to those in the ^1^H-NMR spectra of **2** (around δ 2.79 ppm) and **3** (around δ 2.30 ppm). Their carbonyl carbon resonates significantly up-field (δ_C=O_ 170 ppm) than that of the corresponding precursors **2** (δ_C=O_ 200 ppm) and more downfield than the oxime substrates **3** (δ_C=N_ 154 ppm). The observed significant up-field shift of the methyl protons and the carbonyl carbon of **4a**–**d** compared to **2a**–**d** confirm the formation of the amide derivatives to be the result of an aryl carbon migration. Trifluoroacetic anhydride (TFAA) has previously been found to affect the regioselective Friedel-Crafts trifluoroacetylation of the *N*-benzyl substituted 2-methyl-5-nitroindole [16], the *N*-unprotected 2-arylindoles [17], and their indole-chalcone derivatives [18] at the C-3 position without competitive formation of the 1-acylated and/or the 1,3-diacylated products. A series of fluoromethyl indol-3-yl ketones has also been prepared via Friedel-Craft fluoroacetylation of indoles with fluorinated acetic acids (3 equiv.) in dichloroethane at 100 °C in the absence of catalyst or additive [19]. Compounds **2a–d** and **4a**–**d** were subjected to TFAA (1.2 equiv.) in THF under reflux for 5 h to afford the corresponding 7-acetyl **5a**–**d** and 7-acetamido substituted 5-bromo-3-trifluoroacetylindoles **5e**–**h** in moderate to high yields.

### 2.2. Biological Evaluation

As a prelude to the 3-trifluoroacetyl–substituted 2-arylindole derivatives with potential anticancer properties, we screened compounds **5a**–**h** for in vitro antiproliferative properties against human lung cancer (A549) and cervical cancer (HeLa) cell lines, which have been found to be the main causes of cancer death in males and females worldwide. The corresponding intermediates, namely, the 3-unsubstituted 7-acetyl-2-aryl-5-bromoindole **2a**–**d** and 7-acetamido-2-aryl-5-bromoindole **4a**–**d** precursors were also included in the assays in order to correlate between both structural variations and cytotoxicity.

#### 2.2.1. In Vitro Cytotoxicity Studies of Indole Derivatives **2a**–**d**, **4a**–**d** and **5a**–**h**

The HeLa and A549 cancer cells were initially exposed to two concentrations of each test compound (10 μM and 100 μM) for 48 h with a well-known chemotherapeutic drug, Melphalan, used as a reference standard at the same concentrations (Table 2 and Table 3). Acquisition was performed using the ImageXpress Micro XLS Widefield Microscope and the acquired images were analyzed using the MetaXpress software and Multi-Wavelength Cell Scoring Application Module (see Figure S2 in the Supplementary Information for cell viability percentages for each compound). The structure activity relationship (SAR) of these compounds has been evaluated with respect to the substitution patterns on the 2-phenyl ring, C-3 and C-7 positions. The acetyl derivatives **2a**–**d** were found to be generally inactive against the two cancer cell lines at both concentrations (Table 2). The presence of an acetamido moiety at the 7-position, on the other hand, led to modest cytotoxicity against the two cell lines for the 2-(4-fluorophenyl)- **4b**, 2-(3-chlorophenyl)- **4c** and 2-(4-methoxyphenyl)-substituted 7-acetamido-5-bromoindole **4d** (Table 2). The presence of the amide moiety on the fused benzo ring has previously been found to enhance the biological activity of indole-based compounds [6,10,12]. Lack of cytotoxicity for the 2-phenyl substituted derivative **4a** against both cancer cell lines, on the other hand, presumably reflects the importance of a lipophilic substituent (halogen or methoxy group) on the 2-phenyl ring of compounds **4b**–**d** on biological activity.

Diminished activity against both cell lines was observed for all the 2-aryl-5-bromoindoles **5a**–**d** bearing a combination of the 7-acetyl and 3-trifluoroacetyl groups (Table 3). A combination of a 7-acetamido and 3-trifluoroacetyl groups in compounds **5e**–**h**, on the other hand, resulted in increased cytotoxicity against both cancer cell lines and more so than the chemotherapeutic drug, Melphalan, used as a positive control at the same concentrations (see Supplementary Information (Figure S3) for the corresponding dose response curves). Within this series, the trend in IC_50_ values (Table 4) against the lung cancer (A549) cell line is as follows: **5g** (2.72 µM) > **5h** (3.26 µM) > **5f** (5.03 µM) > **5e** (9.94 µM) with the following trend in activity against the cervical cancer (HeLa) cells: **5f** (7.95 µM) > **5g** (8.74 µM) > **5h** (10.72 µM) > **5e** (12.89 µM). These preliminary cytotoxicity results suggest that a combination of the 7-acetamido and 3-trifluoroacetyl group is desirable for cytotoxicity of the 2-aryl-5-bromoindoles against both cancer cell lines. The slight antiproliferative activity observed for the corresponding C-3 unsubstituted 7-acetamido-5-bromoindoles **4a**–**d**, which significantly increased upon incorporation of a 3-trifluoroacetyl in derivatives **5e**–**h** further confirm the importance of a strong hydrogen bond acceptor at this position 3 of the indole framework to facilitate interaction with biological receptors in analogy with the literature precedents [9,10]. The presence of a strong electron-withdrawing trifluoromethyl group (-CF_3_) has previously been found to increase the activity profile of indole derivatives [11,13]. The combined electron-withdrawing and hydrogen bonding effects of the trifluromethyl and carbonyl fragments of the 3-trifluoroacetyl group probably account for the observed increased cytotoxicity of compounds **5e**–**h**.

In vitro studies indicated that indole derivatives such as indole-3-carbinol inhibit cell proliferation, caused cell cycle arrest at the G1 phase and induced apoptosis [15]. Deregulation of apoptosis plays a significant role in the development of cancer [20]. Cytotoxicity, on the other hand, does not define a specific cellular death mechanism (necrosis or apoptosis). We considered the pro-apoptotic properties of indole-3-carbinol [15] and selected compounds **4c** and **5g** as representative examples for further evaluation regarding the mechanism of action of the 3-trifluoroacetylated 2-arylindole derivatives **5e**–**h** in the A549 and HeLa cells.

#### 2.2.2. Evaluation of Cell Death Pathways

**Figure 2:**
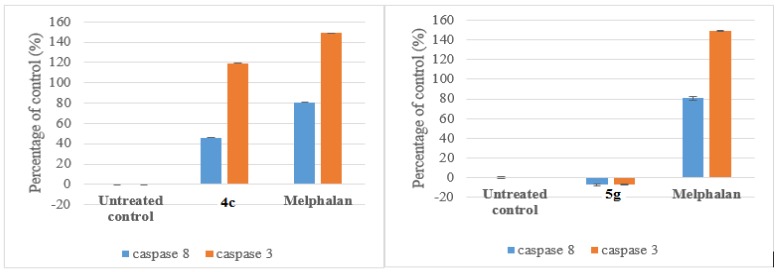
Caspase 8 and 3 activation as indicated by % changes in the mean integrated fluorescence intensity of the untreated control using HeLa cells after 24 h of exposure to compounds **4c** and **5g** (IC_50_ values) using Melphalan as a positive control. Bar graph represents the average of one individual experiment performed in quadruplicate. The standard deviations are represented as error bars.

**Figure 3:**
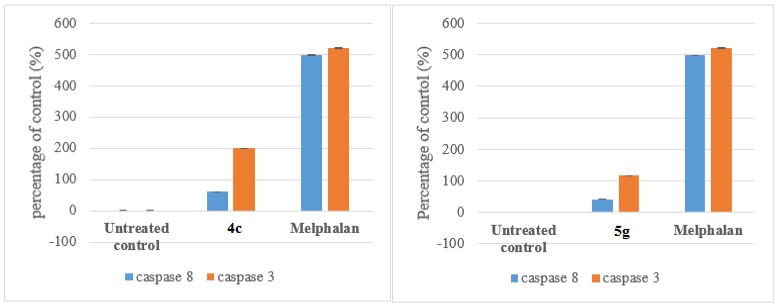
Caspase 8 and 3 activation as indicated by % changes in the mean integrated fluorescence inensity of the untreated control using A549 cells after 24 h of exposure to **4c** and **5g** (IC_50_ values) using Melphalan as a positive control. Bar graph represents the average of one individual experiment performed in quadruplicate. The standard deviations are represented as error bars.

Several distinctive mechanisms of cancer cell death such as apoptosis, necrosis, autophagy and cornification have been identified, and these are characterized by differences in morphology and biochemical changes [21]. A major biochemical feature of apoptosis is the activation of caspases, which plays a central role in the morphological changes associated with apoptosis [21,22,23]. Caspase activation was determined using immunochemical methods and the antibodies against activated caspase 8 and caspase 3. As an initiator caspase, caspase 8 is first activated through a death signal suggesting a cellular response to an external signal for cell death. Activated caspase 8 can be used as a convenient indicator of involvement of the extrinsic death receptor pathway of apoptosis [24]. Executioner caspases including caspase 3 are, in turn, activated by the initiator caspases. This protein is either partially or totally responsible for the cleavage of many key proteins such as PARP, DNA protein kinases and retinoblastoma protein [25]. Figure 2 and Figure 3 show the results of caspase activation analysis and it is evident that clear increases in the percentage of mean fluorescence intensity of the untreated control occurred when the HeLa and A549 cells were treated with **4c** and Melphalan. A decrease in percentage was noticed when HeLa cells were treated with **5g**, but the expected increase was evident in A549 cells. This was true for both activated caspase 8 and 3. A more distinct increase was evident with caspase 3 albeit less pronounced than that of **4c** and the increase was expected as caspase 3 is activated by initiator caspase including caspase 8, which is a later event in apoptosis. It is evident from the observed results that **5g** induces apoptosis in a caspase-dependent manner in both cancer cell lines and this effect is more pronounced for the A549 cells, which also showed increased sensitivity towards this compound in the cytotoxicity assays than the HeLa cells. Prolonged treatment of the cells with compounds **5e**–**h** for 48 h or more, for example, would probably result in significant effects. We also evaluated compound **5g** for potential to induce other key apoptotic biochemical features such as phosphatidylserine (PS) translocation, cell cycle arrest and mitochondrial membrane depolarization. However, the compound failed to induce PS translocation, cell cycle arrest and mitochondrial membrane depolarization in both cell lines after 24 h (data not included).

Recourse to the literature revealed that a series of analogous indole-bearing combretastatin derivatives prepared by condensing indole-3-acetic acid with aldehydes prevent tubulin polymerization as confirmed by immunofluorescence confocal microscopy [26]. Molecular docking revealed that the compounds bind to the colchicine binding site which is situated at α and β interface of tubulin [26]. This literature precedent encouraged us to evaluate the 3-trifluoroacetyl substituted derivatives **5e**–**h** for potential to inhibit tubulin polymerization.

### 2.3. Effect of Compounds ***5e**–**h*** on Tubulin Polymerization

**Figure 4:**
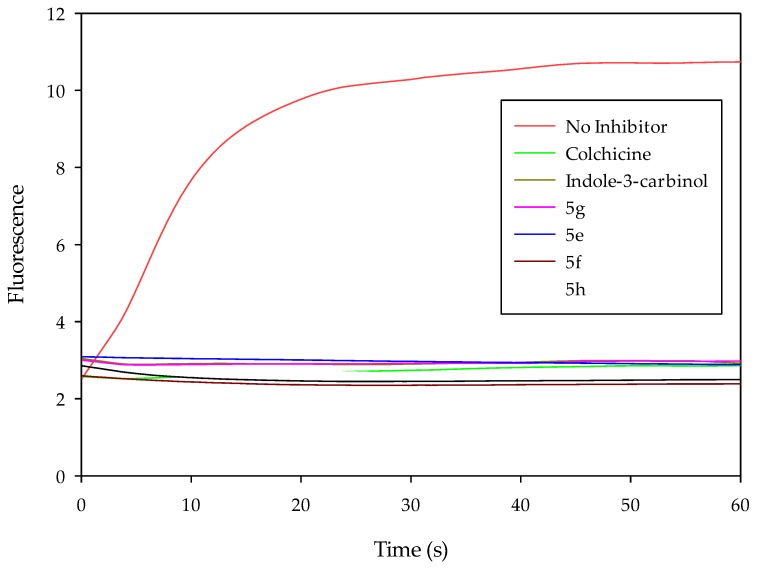
Kinetics of inhibition of tubulin polymerization by **5e**–**h** at 0.25 µM against colchicine (green) and indole-3-carbinol (yellow) and in the absence of an inhibitor (red). Fluorescence at 450 nm was measured every three seconds over a period of 1 h at 37 °C.

In order to determine if the antiproliferative activity of compounds **5e**–**h** was related to their capacity to destabilize tubulin, we evaluated them in a tubulin polymerization assay using colchicine and indole-3-carbinol as reference standards. Kinetics of inhibition of tubulin polymerization of compounds **5e**–**h** at 0.25 µM against colchicine and indole-3-carbinol as reference standards (Figure 4) revealed that these compounds significantly interfere with tubulin polymerization by lowering the rate of microtubule assembly. The IC_50_ values for compounds **5a**–**e** and the corresponding reference standards (Table 3) were obtained at the following concentrations: 0.1, 1.0 and 10 µM. These results are in agreement with the antiproliferative activity in the cell culture.

The increased cytotoxicity and inhibition of tubulin polymerization of compounds **5e**–**h** are probably due to strong hydrogen bond interactions between the trifluoroacetyl group of the ligands with the receptor’s protein residues. The propensity of the amide moiety for hydrogen bonding cannot be over ruled because it plays an important role in the interaction of bioactive compounds with the receptors [27]. In order to prove these assumptions and further ass sit us to rationalize the structure activity relationship (SAR), we subjected the most active compound **5g** to molecular docking into the colchicine-binding site of tubulin.

### 2.4. Molecular Docking Studies of ***2a***, ***4a*** and ***5g*** into Tubulin

**Figure 5:**
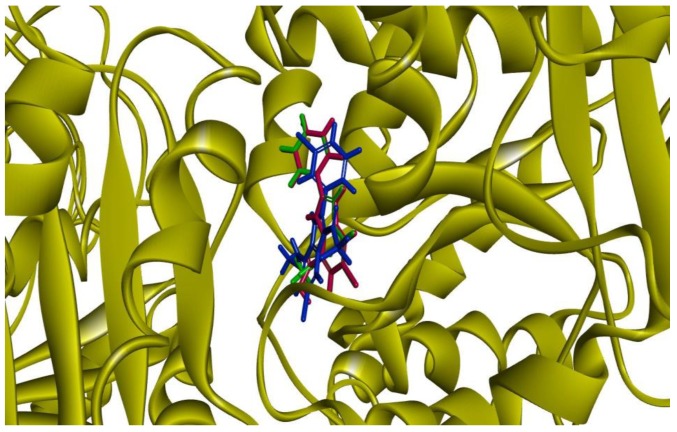
Compounds **2a** (green), **4a** (pink) and **5g** (blue) were docked at the interface of a tubulin heterodimer (PDB code: 1TUB), which is coloured yellow.

To help us understand the anticancer activity of the 3-trifluoroacetyl indole derivatives **5e**–**h** and further rationalize SAR, we docked **5g** and the corresponding parent compounds **2a** and **4a** into tubulin (Figure 5). The crystal structure of tubulin employed in this investigation was obtained from the protein data bank (PDB ID: 1TUB). Compounds **2a**, **4a** and **5g** were docked between the two subunits of the tubulin heterodimer as shown in Figure 5.

**Figure 6:**
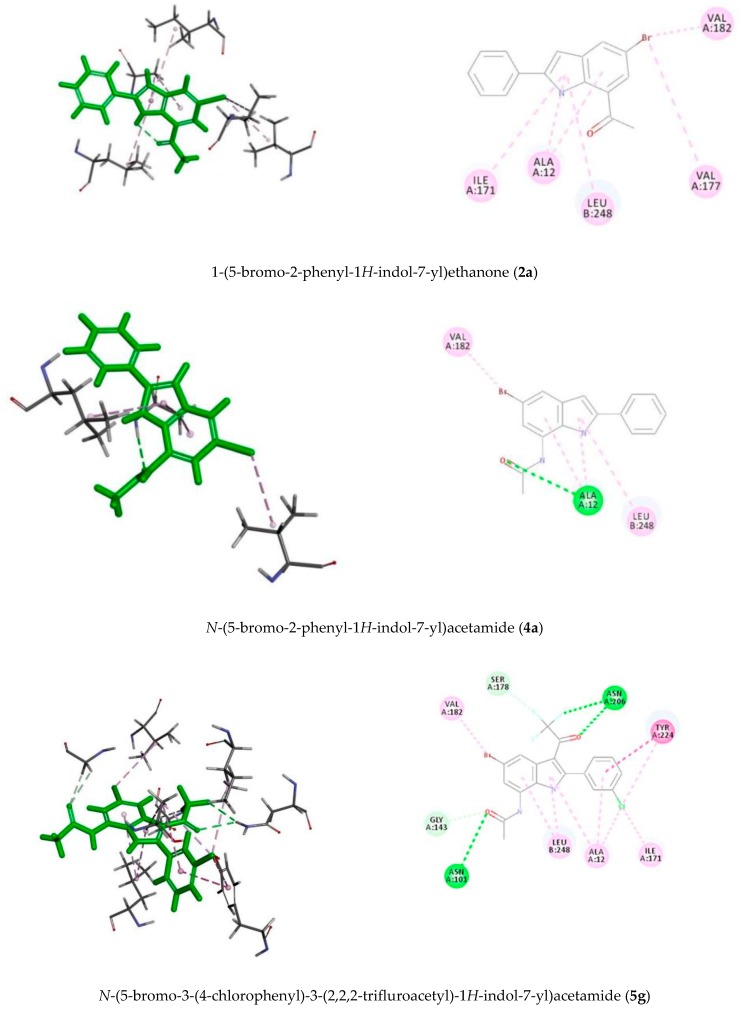
2D interaction diagrams for the binding of tubulin (PDB code 1TUB) with **2a**, **4a** and **5g**. Residues are annotated with their 3-letter amino acid code The various interactions are annotated by color; alkyl/pi-alkyl (pink), carbon hydrogen bond (light green) and conventional hydrogen bond.

Figure 6 reveals the presence of pi-alkyl, amide-pi stacked and alkyl interactions between these aromatic compounds and the active site residues, for example, Ala12, Val177, Val182, Leu248, Ile171 and Tyr224. There is no hydrogen bonding between the acetyl group of **2a** and any of the protein residues. The results of molecular docking and cytotoxicity evaluation against the two cancer cell lines seem to suggest that an acetyl group at the 7-position of the fused benzo ring is less desirable for cytotoxicity. However, the presence of a carbonyl (formyl or acetyl) group was previously found to enhance the antiproliferative properties of the indoles when attached to the C-3 position [9]. Molecular docking of **4a** into the colchicine binding site, on the other hand, revealed a hydrogen bond between the amide functionality and the protein residue Ala12. The propensity of the amide moiety for hydrogen bonding plays a significant role in the interaction of bioactive compounds with the receptors [27] and this may account for the observed slight cytotoxicity of compounds **4b**–**d** against the two cancer cell lines when compared to **2a**–**d**. A combination of the hydrophobic and hydrogen bond interactions are responsible for the strong binding of compound **5g** in the colchicine-binding site of tubulin between the two heterodimers [28]. The presence of the trifluoroacetyl group in **5g** resulted in hydrogen bonding between the carbonyl group (2.6 Å) and fluorine atoms (3.5 Å) with amide group of the amino acid residue, Asn20. Additional hydrogen bond interaction is observed between the acetamido group of compound **5g** and the amide group of Asn101 (2.5 Å) with π-π stacking interaction between the protein residue Tyr224 and the 3-chlorophenyl group. These interactions probably help to stabilize the binding of compounds **5a**–**h** in the colchicine-binding domain of α,β-tubulin interface. A 3-trifluoroacetyl moiety in the 7-acetamido-2-aryl-5-bromoindoles **5e**–**h** in our view enhances the binding of these compounds to tubulin in the colchicine-binding domain of α,β-tubulin interface Such binding would probably lead to cancer cell death (apoptosis).

## 3. Materials and Methods

### 3.1. General

The melting point values of the prepared compounds were recorded on a Thermocouple digital melting point apparatus and are uncorrected. Their IR spectra were recorded as powders on a Bruker VERTEX 70 FT-IR Spectrometer (Bruker Optics, Billerica, MA, USA) with a diamond ATR (attenuated total reflectance) accessory by using the thin-film method. Merck kieselgel 60 (0.063–0.200 mm) (Merck KGaA, Frankfurt, Germany) was used as stationary phase for column chromatography. The ^1^H-NMR and ^13^C-NMR spectra were obtained as DMSO-*d*_6_ solutions using Agilent 300 MHz NMR (Agilent Technologies, Oxford, UK) spectrometer and the chemical shifts are quoted relative to the TMS peak. The high-resolution mass spectra were recorded at an ionization potential of 70 eV using Waters Synapt G2 Quadrupole Time-of-flight mass spectrometer (Waters Corp., Milford, MA, USA) at the University of Stellenbosch Central Analytical Facility (CAF). The synthesis and analytical data of compounds **1a**–**d** have been described before [14].

### 3.2. Typical Procedure for the PdCl_2_-Mediated Heteroannulation of ***1a**–**d***

A stirred mixture of **1** (1 equiv.) and PdCl_2_ (0.2 equiv.) in acetonitrile (15 mL/mmol of **1**) was heated at 80 °C under an argon atmosphere for 3 h. The mixture was evaporated to dryness on a rotary evaporator and the residue was dissolved in chloroform. The organic solvent was washed with brine and then dried over anhydrous MgSO_4_. The salt was filtered off and the solvent was evaporated under reduced pressure on a rotary evaporator. The residue was purified by column chromatography on a silica gel using 10% EtOAc-hexane as eluent to afford **2** as a solid. Compounds **2a**–**d** were prepared in this fashion.

*1-(5-Bromo-2-phenyl-1H-indol-7-yl)ethanone* (**2a**). A mixture of **1a** (1.00 g, 3.18 mmol) and PdCl_2_ (0.11 g, 0.64 mmol) in acetonitrile (50 mL) afforded **2a** as a solid (0.92 g, 92%), *R_f_* 0.36, mp. 139–142 °C; ν_max_ (ATR) 550, 596, 671, 750, 849, 961, 1043, 1163, 1230, 1251, 1322, 1360, 1448, 1490, 1516, 1595, 1650, 3436 cm^−1^; ^1^H-NMR: 2.71 (3H, s, CH_3_), 6.98 (1H, s, 3-H), 7.38 (1H, t, *J* = 7.5 Hz, 4′-H), 7.47 (2H, t, *J* = 7.5 Hz, 3′,5′-H), 7.89 (2H, d, *J* = 7.5 Hz, 2′,6′-H), 7.94 (1H, d, *J* = 1.2 Hz, 4-H), 8.05 (1H, d, *J* = 1.2 Hz, 6-H), 11.14 (1H, s, NH); ^13^C-NMR: 27.6, 99.5, 111.7, 122.1, 126.4, 127.4, 128.6, 128.9, 129.4, 131.3, 132.6, 134.0, 141.3, 199.2; HRMS (ES): found 314.0191. C_16_H_13_NO^79^Br^+^ requires 314.0181. *Anal* calcd for C_16_H_12_NOBr: C, 61.17; H, 3.85; N, 4.46. Found: C, 61.02; H, 3.79; N, 4.52.

*1-[5-Bromo-2-(4-fluorophenyl)-1H-indol-7-yl]ethanone* (**2b**). A mixture of **1b** (1.00 g, 3.01 mmol) and PdCl_2_ (0.11 g, 0.60 mmol) in acetonitrile (50 mL) afforded **2b** as a solid (0.84 g, 84%), *R_f_* 0.35, mp. 171–173 °C; ν_max_ (ATR) 549, 749, 786, 835, 925, 1006, 1043, 1162, 1231, 1251, 1321, 1360, 1449, 1491, 1578, 1595, 1650, 3436 cm^−1^; ^1^H-NMR: 2.69 (3H, s, CH_3_), 6.91 (1H, s, 3-H), 7.29 (2H, t, *J* = 8.7 Hz, 3′,5′-H), 7.91–7.95 (3H, m, 4-H and 2′,6′-H), 8.01 (1H, d, *J* = 1.2 Hz, 6-H), 11.19 (1H, s, NH); ^13^C-NMR: 27.6, 99.4, 111.7, 116.2 (d, ^2^*J*_CF_ = 21.8 Hz), 122.2, 127.3, 128.0 (d, ^4^*J*_CF_ = 3.5 Hz), 128.5, 128.8 (d, ^3^*J*_CF_ = 8.0 Hz), 132.5, 134.0, 140.5, 162.2 (d, ^1^*J*_CF_ = 243.9 Hz), 199.1; HRMS (ES) found 332.0090. C_16_H_12_NO^79^FBr^+^ requires 332.0086. *Anal* calcd for C_16_H_11_NOFBr: C, 57.85; H, 3.34; N, 4.22. Found: C, 57.79; H, 3.30; N, 4.12.

*1-[5-Bromo-2-(3-chlorophenyl)-1H-indol-7-yl]ethanone* (**2c**). A mixture of **1c** (1.00 g, 2.87 mmol) and PdCl_2_ (0.10 g, 0.57 mmol) in acetonitrile (50 mL) afforded **2c** as a solid (0.77 g, 77%), *R_f_* 0.34, mp. 140–143 °C; ν_max_ (ATR) 452, 542, 572, 673, 751, 785, 852, 878, 969, 1052, 1082, 1164, 1252, 1323, 1357, 1452, 1590, 1653, 3424 cm^−1^; ^1^H-NMR: 2.69 (3H, s, CH_3_), 7.04 (1H, s, 3-H), 7.38–7.49 (2H, m, 4′,5′-H), 7.82–7.86 (1H, d, *J* = 8.7 Hz, 6′-H), 7.93 (1H, d, *J* = 1.2 Hz, 4-H), 8.03–8.04 (2H, m, 2′-H and 6-H), 11.33 (1H, s, NH); ^13^C-NMR: 27.6, 100.7, 111.8, 122.4, 125.2, 126.2, 127.8, 128.4, 128.7, 131.0, 132.3, 133.4, 134.0, 134.1, 139.8, 198.9; HRMS (ES): found 347.9779. C_16_H_12_NO^35^Cl^79^Br^+^ requires 347.9791. *Anal* calcd for C_16_H_11_NOClBr: C, 55.12; H, 3.18; N, 4.02. Found: C, 55.10; H, 3.33; N, 3.89.

*1-[5-Bromo-2-(4-methoxyphenyl)-1H-indol-7-yl]ethanone* (**2d**). A mixture of **1d** (1.00 g, 2.91 mmol) and PdCl_2_ (0.10 g, 0.58 mmol) in acetonitrile (50 mL) afforded **2d** as a solid (0.85 g, 85%), *R_f_* = 0.27, mp. 140–142 °C; ν_max_ (ATR) 550, 589, 749, 825, 925, 1028, 1162, 1181, 1253, 1290, 1321, 1361, 1451, 1497, 1578, 1654, 3433 cm^−1^; ^1^H-NMR: 2.69 (3H, s, CH_3_), 3.80 (3H, s, OCH_3_), 6.86 (1H, s, 3-H), 7.03 (2H, d, *J* = 8.7 Hz, 3′,5′-H), 7.84 (2H, d, *J* 8.7 Hz, 2′,6′-H), 7.90 (1H, d, *J* = 1.2 Hz, 4-H), 8.01 (1H, d, *J* = 1.2 Hz, 6-H), 11.06 (1H, s, NH); ^13^C-NMR: 27.6, 55.7, 98.2, 111.6, 114.8, 121.9, 123.8, 126.8, 127.9, 128.2, 132.8, 133.9, 141.5, 160.0, 199.3; HRMS (ES) found 344.0289. C_17_H_15_NO_2_^79^Br^+^ requires 344.0286. *Anal* calcd for C_17_H_14_NO_2_Br: C, 59.32; H, 4.10; N, 4.07. Found: C, 59.32; H, 3.87; N, 4.10.

### 3.3. Typical Procedure for the Synthesis of Oxime Derivatives ***3a**–**d*** from ***2a**–**d***

A stirred mixture of **2** (1 equivalent), hydroxylamine hydrochloride (1.5 equivalent) and pyridine (1.5 equivalent) in ethanol (20 mL/mmol of **2**) was heated at 80 °C for 5 h. The mixture was cooled to room temperature, quenched with an ice-cold water and then extracted with chloroform. The combined organic phases were washed with water and dried over anhydrous MgSO_4_. The salt was filtered off and the solvent was evaporated under reduced pressure on a rotary evaporator. The residue was purified by column chromatography on silica gel using 20% EtOAc-hexane as an eluent to afford the oxime derivative **3** as a solid. Products **3a**–**d** were prepared in this fashion:

*1-(5-Bromo-2-phenyl-1H-indol-7-yl)ethanone oxime* (**3a**). A mixture of **2a** (0.30 g, 0.95 mmol), hydroxylamine hydrochloride (0.10 g, 1.43 mmol) and pyridine (0.11 g, 1.43 mmol) in ethanol (20 mL) afforded **3a** as a solid (0.28 g, 90%), *R_f_* = 0.69, mp. 198–200 °C; ν_max_ (ATR) 524, 542, 634, 679, 734, 763, 839, 989, 1093, 1172, 1261, 1298, 1331, 1368, 1448, 3269, 3438 cm^−1^; ^1^H-NMR: 2.30 (3H, s, CH_3_), 6.96 (1H, s, 3-H), 7.37 (1H, t, *J* = 7.5 Hz, 4′-H), 746–7.52 (3H, m, 6-H and 3′,5′-H), 7.78–7.81 (3H, m, 4-H and 2′,6′-H), 10.94 (1H, s, NH), 11.56 (1H, s, OH); ^13^C-NMR: 11.2, 99.3, 112.4, 121.7, 123.4, 123.8, 125.5, 128.7, 129.6, 131.3, 131.5, 132.5, 139.2, 154.1; HRMS (ES): found 329.0276. C_16_H_14_N_2_O^79^Br^+^ requires 329.0290.

*1-(5-Bromo-2-(4-fluorophenyl)-1H-indol-7-yl)ethanone oxime* (**3b**). A mixture of **2b** (0.30 g, 0.90 mmol), hydroxylamine hydrochloride (0.09 g, 1.35 mmol) and pyridine (0.11 g, 1.35 mmol) in ethanol (20 mL) afforded **3b** as a solid (0.27 g, 87%), *R_f_* = 0.64, mp. 213–216 °C; ν_max_ (ATR) 530, 595, 651, 680, 744, 764, 828, 846, 912, 984, 1091, 1159, 1230, 1332, 1366, 1428, 1464, 1501, 3258, 3432 cm^−1^; ^1^H-NMR: 2.30 (3H, s, CH_3_), 6.93 (1H, s, 3-H), 7.34 (2H, t, *J* = 8.7 Hz, 3′,5′-H), 7.45 (1H, d, *J* = 1.2 Hz, 6-H), 7.77 (1H, d, *J* = 1.2 Hz, 4-H), 7.84–7.87 (2H, dd, *J* = 5.4 and 8.7 Hz, 2′,6′-H), 10.92 (1H, s, NH), 11.56 (1H, s, OH); ^13^C-NMR: 11.3, 99.3, 112.5, 116.5 (d, ^2^*J*_CF_ = 21.8 Hz), 121.8, 123.4, 123.7, 127.7 (d, ^3^*J*_CF_ = 9.1 Hz), 128.3 (d, ^4^*J*_CF_ = 3.5 Hz), 131.3, 132.5, 138.4, 154.0, 162.4 (d, ^1^*J*_CF_ = 243.9 Hz); HRMS (ES): found 347.0179. C_16_H_13_N_2_OF^79^Br^+^ requires 347.0195.

*1-(5-Bromo-2-(3-chlorophenyl)-1H-indol-7-yl)ethanone oxime* (**3c**). A mixture of **2c** (0.30 g, 0.86 mmol), hydroxylamine hydrochloride (0.09 g, 1.29 mmol) and pyridine (0.10 g, 1.29 mmol) in ethanol (20 mL) afforded **3c** as a solid (0.26 g, 82%), *R_f_* = 0.63, mp. 204–206 °C; ν_max_ (ATR) 454, 532, 631, 673, 742, 784, 847, 876, 947, 985, 1094. 1167, 1268, 1268, 1296, 1334, 1368, 1421, 1463, 3203, 3392 cm^−1^; ^1^H-NMR: 2.32 (3H, s, CH_3_), 7.09 (1H, s, 3-H), 7.44–7.55 (3H, m, 6-H and 4′,5′-H), 7.78–7.81 (2H, m, 4-H and 6′-H), 7.92 (1H, s Hz, 2′-H), 11.02 (1H, s, NH), 11.60 (1H, s, OH); ^13^C-NMR: 11.5, 100.5, 112.6, 122.1, 123.8, 123.9, 124.2, 125.1, 128.3, 131.1, 131.4, 132.7, 133.4, 134.4, 137.7, 153.8; HRMS (ES): found 362.9893. C_16_H_13_N_2_O^35^Cl^79^Br^+^ requires 362.9900.

*1-(5-Bromo-2-(4-methoxyphenyl)-1H-indol-7-yl)ethanone oxime* (**3d**). A mixture of **2d** (0.30 g, 0.87 mmol), hydroxylamine hydrochloride (0.09 g, 1.31 mmol) and pyridine (0.10 g, 1.31 mmol) in ethanol (20 mL) afforded **3d** as a solid (0.25 g, 80%), *R_f_* = 0.45, mp. 202–204 °C; ν_max_ (ATR) 521, 585, 612, 653, 671, 755, 796, 826, 984, 1017, 1172, 1185, 1246, 1323, 1372, 1439, 1460, 1497, 3372, 3518 cm^−1^; ^1^H-NMR: 2.30 (3H, s, CH_3_), 3.80 (3H, s, OCH_3_), 6.80 (1H, s, 3-H), 7.04 (2H, d, *J* = 8.7 Hz, 3′,5′-H), 7.42 (1H, d, *J* = 1.2 Hz, 6-H), 7.70 (3H, m, 4-H and 2′,6′-H), 10.84 (1H, s, NH), 11.57 (1H, s, OH); ^13^C-NMR: 11.3, 55.7, 98.0, 112.3, 115.0, 121.4, 122.9, 123.4, 124.2, 126.9, 131.6, 132.2, 139.4, 154.2, 159.8; HRMS (ES): found 359.0293. C_17_H_16_N_2_O_2_^79^Br^+^ requires 359.0395.

### 3.4. Typical Procedure for the Beckmann Rearrangement of ***3a**–**d***

A stirred mixture of **3** (1 equiv.) and TFA (1.2 equiv.) in acetonitrile (10 mL/mmol of **3**) was heated at 80 °C for 2 h. The mixture was cooled to room temperature, quenched with ice-cold water and the product was extracted with chloroform (3 × 20 mL). The combined organic layers were dried over anhydrous MgSO_4_ and the salt was filtered off. The solvent was evaporated under reduced pressure on a rotary evaporator and the residue was purified by column chromatography on a silica gel using 60% EtOAc-hexane as an eluent to afford **4**. The following compounds were prepared in this fashion:

*N-(5-Bromo-2-phenyl-1H-indol-7-yl)acetamide* (**4a**). A mixture of **3a** (0.20 g, 0.61 mmol) and TFA (0.08 g, 0.73 mmol) in acetonitrile (15 mL) afforded **4a** as solid (0.16 g, 81%), *R_f_* = 0.56, mp. 243–244 °C; ν_max_ (ATR) 518, 562, 600, 688, 740, 760, 842, 998, 1184, 1273, 1315, 1404, 1425, 1455, 1530, 1623, 1651, 3267, 3319 cm^−1^; ^1^H-NMR: 2.19 (3H, s, CH_3_), 6.88 (1H, s, 3-H), 7.38 (1H, t, *J* = 7.5 Hz, 4′-H), 7.46–7.52 (3H, m, 4-H and 3′,5′-H), 7.82 (2H, d, *J* = 7.5 Hz, 2′,6′-H), 7.88 (1H, d, *J* = 1.2 Hz, 6-H), 9.76 (1H, s, NH), 11.28 (1H, s, NH); ^13^C-NMR: 24.5, 99.5, 112.1, 115.5, 118.2, 125.5, 125.7, 127.3, 128.5, 129.5, 131.6, 131.9, 139.0, 169.1; HRMS (ES): found 329.0265. C_16_H_14_N_2_O^79^Br^+^ requires 329.0289. *Anal* calcd for C_16_H_13_N_2_OBr: C, 58.38; H, 3.98; N, 8.51. Found: C, 58.24; H, 3.93; N, 8.47.

*N-[5-Bromo-2-(4-fluorophenyl)-1H-indol-7-yl]acetamide* (**4b**). A mixture of **3b** (0.20 g, 0.58 mmol) and TFA (0.08 g, 0.69 mmol) in acetonitrile (15 mL) afforded **4b** as solid (0.14 g, 76%); *R_f_* = 0.53, mp. 263–265 °C; ν_max_ (ATR) 519, 576, 695, 744, 780, 837, 1000, 1163, 1231, 1274, 1318, 1438, 1474, 1502, 1542, 1624, 1654, 3267, 3355 cm^−1^; ^1^H-NMR: 2.17 (3H, s, CH_3_), 6.84 (1H, s, 3-H), 7.34 (2H, t, *J* = 8.7 Hz, 3′,5′-H), 7.44 (1H, d, *J* = 1.2 Hz, 4-H), 7.82–7.86 (3H, m, 6-H and 2′,6′-H), 9.71 (1H, s, NH), 11.23 (1H, s, NH); ^13^C-NMR: 24.5, 99.4, 112.2, 115.7, 116.5 (d, ^2^*J*_CF_ = 21.8 Hz), 118.3, 125.4, 127.5, 127.8 (d, ^3^*J*_CF_ = 8.0 Hz), 128.6 (d, ^4^*J*_CF_ = 3.4 Hz), 131.5, 138.1, 162.3 (d, ^1^*J*_CF_ = 243.9 Hz), 169.1; HRMS (ES): found 347.0168. C_16_H_13_N_2_OF^79^Br^+^ requires 347.0195. *Anal* calcd for C_16_H_12_N_2_OFBr: C, 55.35; H, 3.48; N, 8.07. Found: C, 55.22; H, 3.39; N, 7.89.

*N-(5-Bromo-2-(3-chlorophenyl)-1H-indol-7-yl)acetamide* (**4c**). A mixture of **3c** (0.30 g, 0.55 mmol) and TFA (0.08 g, 0.66 mmol) in acetonitrile (15 mL) afforded **4c** as solid (0.15 g, 73%), *R_f_* = 0.54, mp. 261–263 °C; ν_max_ (ATR) 562, 598, 668, 680, 751, 775, 846, 1010, 1095, 1277, 1317, 1445, 1462, 1538, 1573, 1622, 1650, 3268, 3397 cm^−1^; ^1^H-NMR: 2.18 (3H, s, CH_3_), 6.97 (1H, s, 3-H), 7.38–7.53 (3H, m, 4-H and 4′,5′-H), 7.77 (1H, d. *J* = 8.7 Hz, 6′-H), 7.87–7.90 (2H, m, 6-H and 2′-H), 9.70 (1H, s, NH), 11.28 (1H, s, NH); ^13^C-NMR: 24.6, 100.6, 112.3, 116.2, 118.5, 124.4, 125.0, 125.5, 127.8, 128.1, 131.3, 131.4, 134.0, 134.4, 137.3, 169.1; HRMS (ES): found 362.9890. C_16_H_13_N_2_O^35^Cl^79^Br^+^ requires 362.9900. *Anal* calcd for C_16_H_12_N_2_OClBr: C, 52.85; H, 3.33; N, 7.70. Found: C, 52.78; H, 3.41; N, 7.59.

*N*-[5-Bromo-2-(4-methoxyphenyl)-1*H*-indol-7-yl]acetamide (**4d**). A mixture of **3d** (0.30 g, 0.56 mmol) and TFA (0.08 g, 0.67 mmol) in acetonitrile (15 mL) afforded **4d** as solid (0.16 g, 76%), *R_f_* = 0.36, mp. 226–228 °C; ν_max_ (ATR) 559, 584, 694 786, 834, 1021, 1179, 1244, 1320, 1398, 1439, 1500, 1612, 1658, 3300, 3403 cm^−1^; ^1^H-NMR: 2.18 (3H, s, CH_3_), 3.80 (3H, s, OCH_3_), 6.74 (1H, s, 3-H), 7.06 (2H, d, *J* = 8.7 Hz, 3′,5′-H), 7.41 (1H, d, *J* = 1.2 Hz, 4-H), 7.74 (2H, d, *J* = 8.7 Hz, 2′,6′-H), 7.83 (1H, d, *J* = 1.2 Hz, 6-H), 9.71 (1H, s, NH), 11.14 (1H, s, NH); ^13^C-NMR: 24.6, 55.7, 98.2, 112.0, 114.9 (2C), 115.1, 117.9, 124.5, 125.2, 127.1, 131.8, 139.1, 159.7, 169.1; HRMS (ES): found 359.0393. C_17_H_16_N_2_O_2_^79^Br^+^ requires 359.0395. *Anal* calcd for C_17_H_15_N_2_O_2_Br: C, 56.84; H, 4.21; N, 7.80. Found: C, 56.79; H, 4.13; N, 7.56.

### 3.5. Typical Procedure for the Trifluoroacetylation of ***2a**–**d*** and ***4a**–**d***

A mixture of **2**/**4** (1 equivalent) and TFAA (1.5 equivalent) in THF (15 mL/mmol of **1**/**4**) was heated at 60 °C for 5 h. The mixture was cooled to room temperature and quenched with saturated sodium hydrogen carbonate solution. The mixture was extracted with chloroform (3 × 20 mL) and the combined organic layers were dried with anhydrous MgSO_4_. The salt was filtered off and the solvent was evaporated under reduced pressure on a rotary evaporator. The residue was purified by column chromatography on a silica gel using 20% or 60% EtOAc-hexane as eluent to afford **5a**−**d** or **5e**−**h** as solids, respectively. Compounds **5a**–**h** were prepared in this fashion.

*1-(7-Acetyl-5-bromo-2-phenyl-1H-indol-3-yl)-2,2,2-trifluoroethanone* (**4a**). A mixture of **2a** (0.30 g, 0.95 mmol) and TFAA (0.30 g, 1.43 mmol) in THF (15 mL) afforded **5a** as solid (0.30 g, 78%); *R_f_* 0.86, mp. 175–176 °C; ν_max_ (ATR) 515, 647, 671, 697, 746, 767, 861, 911, 994, 1083, 1143, 1203, 1275, 1358, 1433, 1450, 1660, 3401 cm^−1^; ^1^H-NMR: 2.70 (3H, s, CH_3_), 7.47–7.55 (5H, m, Ph), 8.14 (1H, d, *J* = 1.2 Hz, 4-H), 8.42 (1H, d, *J* = 1.2 Hz, 6-H), 12.52 (1H, s, NH); ^13^C-NMR: 27.8, 107.3, 115.6, 116.2 (q, ^1^*J*_CF_ 288.5 Hz), 123.7, 128.1, 128.3, 129.3, 130.4, 130.5 (2C), 130.7, 132.3, 151.6, 176.6 (q, ^2^*J*_CF_ 35.6 Hz), 198.3; HRMS (ES): found 410.0015. C_18_H_12_NO_2_F_3_^79^Br^+^ requires 410.0003. *Anal* calcd for C_18_H_11_NO_2_F_3_Br: C, 52.71; H, 2.70; N, 3.41. Found: C, 52.62; H, 2.35; N, 3.39.

*1-[7-Acetyl-5-bromo-2-(4-fluorophenyl)-1H-indol-3-yl]-2,2,2-trifluoroethanone* (**5b**). A mixture of **2b** (0.30 g, 0.90 mmol) and TFAA (0.28 g, 1.35 mmol) in THF (15 mL) afforded **5b** as solid (0.26 g, 69%); *R_f_* 0.86, mp. 168–171 °C; ν_max_ (ATR) 518, 587, 645, 667, 706, 746, 842, 911, 999, 1082, 1142, 1205, 1244, 1274, 1435, 1489, 1606, 1662, 3401 cm^−1^; ^1^H-NMR: 2.70 (3H, s, CH_3_), 7.33 (2H, t, *J* 8.5 Hz, 3′,5′-H), 7.62 (2H, t, *J* = 6.9 Hz, 2′,6′-H), 8.14 (1H, d, *J* = 1.2 Hz, 4-H), 8.42 (1H, d, *J* = 1.2 Hz, 6-H), 12.61 (1H, s, NH); ^13^C-NMR: 27.8, 107.4, 115.1 (d, ^2^*J*_CF_ = 21.8 Hz), 115.7, 116.3 (q, ^1^*J*_CF_ = 288.6 Hz), 123.6, 127.1 (d, ^4^*J*_CF_ = 3.5 Hz), 128.4, 129.4, 130.3, 132.2, 133.2 (d, ^3^*J*_CF_ = 8.0 Hz), 150.7, 163.6 (d, ^1^*J*_CF_ = 246.2 Hz), 176.3 (q, ^2^*J*_CF_ = 35.5 Hz), 198.3; HRMS (ES): found 427.9721. C_18_H_11_NO_2_F_4_^79^Br^+^ requires 427.9909. *Anal* calcd for C_18_H_10_NO_2_F_4_Br: C, 50.49; H, 2.35; N, 3.27. Found: C, 50.44; H, 2.31; N, 3.30.

*1-[7-Acetyl-5-bromo-2-(3-chlorophenyl)-1H-indol-3-yl]-2,2,2-trifluoroethanone* (**5c**). A mixture of **2c** (0.30 g, 0.86 mmol) and TFAA (0.27 g, 1.29 mmol) in THF (15 mL) afforded **5c** as solid (0.21 g, 56%); *R_f_* 0.86, mp. 138–141 °C; ν_max_ (ATR) 581, 661, 702, 728, 769, 795, 870, 903, 913, 946, 997, 1074, 1147, 1209, 1281, 1316, 1433, 1462, 1651, 3257 cm^−1^; ^1^H-NMR: 2.70 (3H, s, CH_3_), 7.51–7.52 (2H, m, 4′,5′-H), 7.60–7.62 (1H, m, 6′-H), 7.67 (1H, s, 2′-H), 8.15 (1H, d, *J* = 1.2 Hz, 4-H), 8.42 (1H, d, *J* = 1.2 Hz, 6-H), 12.72 (1H, s, NH); ^13^C-NMR: 27.9, 107.5, 115.7, 116.3 (q, ^1^*J*_CF_ 288.6 Hz), 123.7, 128.4, 129.6, 129.7, 129.9, 130.1, 130.2, 130.5, 132.2, 132.6, 132.7, 149.9, 176.2 (q, ^2^*J*_CF_ 35.5 Hz), 198.3; HRMS (ES): found 443.9623. C_18_H_11_NO_2_F_3_^35^Cl^79^Br^+^ requires 443.9614. *Anal* calcd for C_18_H_10_NO_2_F_3_ClBr: C, 48.62; H, 2.27; N, 3.15. Found: C, 48.47; H, 2.26; N, 2.98.

*1-[7-Acetyl-5-bromo-2-(4-methoxyphenyl)-1H-indol-3-yl]-2,2,2-trifluoroethanone* (**5d**). A mixture of **2d** (0.30 g, 0.87 mmol) and TFAA (0.27 g, 1.31 mmol) in THF (15 mL) afforded **5d** as solid (0.29 g, 75%); *R_f_* 0.70, mp. 170–173 °C; ν_max_ (ATR) 615, 642, 660, 746, 836, 910, 1028, 1082, 1140, 1173, 1202, 1262, 1437, 1489, 1607, 1657, 3392 cm^−1^; ^1^H-NMR: 2.70 (3H, s, CH_3_), 3.83 (3H, s, OCH_3_), 7.05 (2H, d, *J* = 8.7 Hz, 3′,5′-H), 7.52 (2H, d, *J* = 8.7 Hz, 2′,6′-H), 8.11 (1H, d, *J* = 1.2 Hz, 4-H), 8.38 (1H, d, *J* = 1.2 Hz, 6-H), 12.32 (1H, s, NH); ^13^C-NMR: 27.9, 107.0, 113.7, 115.5, 116.3 (q, ^1^*J*_CF_ = 289.7 Hz), 122.8, 123.0, 123.5, 128.2, 129.2, 130.5, 132.2, 132.4, 151.8, 161.2, 176.7 (q, ^2^*J*_CF_ = 35.6 Hz), 198.5; HRMS (ES): found 440.0110. C_19_H_14_NO_3_F_3_^79^Br^+^ requires 440.0109. *Anal* calcd for C_19_H_13_NO_3_F_3_Br: C, 51.84; H, 2.98; N, 3.18. Found: C, 51.78; H, 2.93; N, 3.14.

*N-[5-Bromo-2-phenyl-3-trifluoroacetyl-1H-indol-7-yl]acetamide* (**5e**). A mixture of **4a** (0.30 g, 0.91 mmol) and TFAA (0.29 g, 1.37 mmol) in THF (15 mL) afforded **5e** as solid (0.32 g, 84%); *R_f_* 0.49, mp. 141–144 °C; ν_max_ (ATR) 502, 582, 659, 699, 738, 773, 863, 882, 922, 1010, 1143, 1199, 1269, 1334, 1389, 1434, 1449, 1546, 1627, 1655, 3210, 3329 cm^−1^; ^1^H-NMR: 2.11 (3H, s, CH_3_), 7.53–7.63 (5H, m, Ar), 7.94 (1H, d, *J* = 1.2 Hz, 4-H), 7.96 (1H, d, *J* = 1.2 Hz, 6-H), 9.74 (1H, s, NH), 12.53 (1H, s, NH); ^13^C-NMR: 24.3, 107.2, 116.2, 116.6 (q, ^1^*J*_CF_ = 295.5 Hz), 118.8, 126.2, 126.3, 126.8, 128.6, 129.5, 130.3, 130.6, 131.3, 149.2, 169.3, 175.9 (q, ^2^*J*_CF_ = 35.6 Hz); HRMS (ES): found 425.0107. C_18_H_13_N_2_O_2_F_3_^79^Br^+^ requires 425.0112. *Anal* calcd for C_18_H_12_N_2_O_2_F_3_Br: C, 50.85; H, 2.84; N, 6.99. Found: C, 50.52; H, 2.87; N, 6.94.

*N-[5-Bromo-2-(4-fluorophenyl)-3-trifluoroacetyl-1H-indol-7-yl]acetamide* (**5f**). A mixture of **4b** (0.30 g, 0.86 mmol) and TFAA (0.27 g, 1.30 mmol) in THF (15 mL) afforded **5f** as solid (0.29 g, 78%); *R_f_* 0.50, mp. 195–197 °C; ν_max_ (ATR) 515, 611, 732, 844, 884, 924, 1011, 1044, 1146, 1198, 1224, 1271, 1330, 1447, 1497, 1631, 1659, 3196, 3286 cm^−1^; ^1^H-NMR: 2.12 (3H, s, CH_3_), 7.41 (2H, t, *J* = 9.0 Hz, 3′,5′-H), 7.69 (2H, d, *J* = 8.7 Hz, 2′,6′-H), 7.93 (1H, d, *J* = 1.2 Hz, 4-H), 7.94 (1H, d, *J* = 1.2 Hz, 6-H), 9.77 (1H, s, NH), 12.54 (1H, s, NH); ^13^C-NMR: 24.2, 107.4, 115.7 (d, ^2^*J*_CF_ 21.8 Hz), 116.1, 116.5 (q, ^1^*J*_CF_ 288.6 Hz), 118.7, 118.8, 126.2, 126.9, 127.6 (d, ^4^*J*_CF_ = 3.5 Hz), 129.4, 133.8 (d, ^3^*J*_CF_ = 9.1 Hz), 148.2, 163.6 (d, ^1^*J*_CF_ = 246.2 Hz), 169.3, 176.6 (q, ^2^*J*_CF_ = 35.5 Hz); HRMS (ES): found 443.0020. C_18_H_13_N_2_O_2_F_4_^79^Br^+^ requires 443.0020. *Anal* calcd for C_18_H_12_N_2_O_2_F_4_Br: C, 48.78; H, 2.50; N, 6.32. Found: C, 48.59; H, 2.39; N, 6.30.

*N-[5-Bromo-2-(3-chlorophenyl)-3-trifluoroacetyl-1H-indol-7-yl]acetamide* (**5g**). A mixture of **4c** (0.30 g, 0.83 mmol) and TFAA (0.26 g, 1.23 mmol) in THF (15 mL) afforded **5g** as solid (0.19 g, 52%); *R_f_* 0.50, mp. 172–174 °C; ν_max_ (ATR) 541, 570, 685, 746, 794, 851, 913, 1010, 1039, 1083, 1143, 1198, 1260, 1369, 1441, 1542, 1630, 1660, 3217, 3356 cm^−1^; ^1^H-NMR: 2.12 (3H, s, CH_3_), 7.60–7.66 (3H, m, Ar), 7.75 (1H, s, 2′-H), 8.28 (1H, d, *J* = 1.2 Hz, 4-H), 8.29 (1H, d, *J* = 1.2 Hz, 6-H), 9.77 (1H, s, NH), 12.61 (1H, s, NH); ^13^C-NMR: 24.3, 107.4, 116.2, 116.5 (q, ^1^*J*_CF_ = 289.7 Hz), 118.9, 126.3, 127.2, 129.3, 129.9, 130.5, 133.2, 133.4, 147.3, 169.3, 175.6 (q, ^2^*J*_CF_ = 34.4 Hz); HRMS (ES): found 458.9731. C_18_H_12_F_3_N_2_O_2_^35^Cl^79^Br^+^ requires 458.9723. *Anal* calcd for C_18_H_11_F_3_N_2_O_2_ClBr: C, 47.03; H, 2.41; N, 6.09. Found: C, 47.10; H, 2.36; N, 6.05.

*N-[5-Bromo-2-(4-methoxyphenyl)-3-trifluoroacetyl-1H-indol-7-yl]acetamide* (**5h**). A mixture of **4d** (0.30 g, 0.83 mmol) and TFAA (0.26 g, 1.25 mmol) in THF (15 mL) afforded **5h** as solid (0.30 g, 80%); *R_f_* 0.23, mp. 179–182 °C; ν_max_ (ATR) 520, 573, 643, 751, 838, 884, 922, 1009, 1031, 1154, 1191, 1250, 1415, 1448, 1499, 1538, 1629, 1645, 3211, 3369 cm^−1^; ^1^H-NMR: 2.08 (3H, s, CH_3_), 3.80 (3H, s, OCH_3_), 7.07 (2H, d, *J* = 8.7 Hz, 3′,5′-H), 7.52 (2H, d, *J* = 8.7 Hz, 2′,6′-H), 7.86 (1H, d, *J* = 1.2 Hz, 4-H), 9.91 (1H, d, *J* = 1.2 Hz, 6-H), 9.74 (1H, s, NH), 12.39 (1H, s, NH); ^13^C-NMR: 24.2, 55.8, 107.0, 114.1, 116.0, 116.6 (q, ^1^*J*_CF_ = 289.7 Hz),118.4, 118.6, 123.2, 126.3, 126.8, 129.6, 131.9, 149.5, 161.2, 169.2, 176.9 (q, ^2^*J*_CF_ = 35.5 Hz); HRMS (ES): found 455.0195. C_19_H_15_N_2_O_3_F_3_^79^Br^+^ requires 455.0219. *Anal* calcd for C_19_H_14_N_2_O_3_F_3_Br: C, 50.13; H, 3.10; N, 6.15. Found: C, 49.95; H, 3.20; N, 6.00.

### 3.6. Evaluation for Cytotoxicity

#### 3.6.1. Screening Protocol

The human cervical cancer cell line, HeLa, and lung cancer cell line, A549 were used for the screening assay. HeLa cells were grown and maintained in RPMI 1640 medium supplemented with 10% fetal bovine serum (FBS) and A549 cells were grown and maintained in EMEM supplemented with 10% FBS and 10% non-essential amino acids. In the case of screening experiments, the cells were seeded into 96 well microtiter plates at a density of 6000 cells/well to a total volume of 200 μL per well. The microtiter plates were incubated at 37 °C, 5% CO_2_, and 100% relative humidity for 24 h to allow for cell attachment. Each of the test compounds was first reconstituted in DMSO at a concentration of 40 mM and then stored at 4 °C prior to use and final dilution to working concentrations with culture complete medium on the day of treatment. Two hundred microliters aliquots of the diluted compound in fresh medium was used to treat cells after aspiration of seeding medium. Each of the cell lines was incubated for 48 h at 37 °C in a humidified incubator under 5% CO_2_ atmosphere. Treatment medium was aspirated from all wells and replaced with 100 μL of Hoechst 33342 nuclear dye (5 μg/mL) and incubated for 10 min at room temperature. Thereafter, cells were stained with propidium iodide (PI) at 100 μg/mL in order to enumerate the proportion of dead cells within the population. Cells were imaged immediately after addition of PI using the ImageXpress Micro XLS Widefield Microscope (Separations, Randburg, South Africa).

#### 3.6.2. Dose Response Analysis and Determination of IC_50_ Values

Cells were seeded as described in above. The compounds were tested at a concentration range of 0–100 μM to determine their respective IC_50_ values on lung cancer (A549) cells and cervical cancer (HeLa) cells. The medium was aspirated and replaced with 200 μL fresh medium containing the dilution of the respective test compound. The test sample were added to each well and the plates were incubated for a further 48 h at 37 °C under 5% CO_2_ atmosphere in a humidified incubator. After this incubation period, the medium was removed and then replaced with 100 μL fresh culture medium containing MTT at a final concentration of 0.5 mg/mL. The 96-well plates were returned to the incubator and incubated for an additional 3 h. The medium was removed and the MTT crystals were solubilized in 100 μL DMSO and the absorbance was measured at 560 nm using a Labsystems Multiwell Scanning spectrophotometer (Thermo Fischer Scientific, Edenvale, South Africa).

#### 3.6.3. Data Analysis

Quantification of live and dead cells for the screening assay was performed using the ImageXpress Micro XLS Widefield Microscope and acquired images analysed using the MetaXpress software and Multi-Wavelength Cell Scoring Application Module. Acquired data was transferred to an EXCEL spreadsheet and relative cell viability was determined using quadruplicate wells for each concentration. Untreated cells were considered to have 100% cell viability (i.e., the mean OD of the untreated wells = 100% viability). Cell viabilities in other test wells were calculated relative to the untreated control and expressed as a percentage. Dose response analysis was performed using the statistical software GraphPad Prism and IC_50_ values calculated from the concentration-response data using a mathematical Hill function. If the concentration range selected did not produce 100% inhibition, a lower concentration equal to zero was included to allow IC_50_ determination.

### 3.7. Evaluation of Mode of Cell Death

#### 3.7.1. Caspace Activation

Compounds **4c** and **5g** were selected for mechanism studies and these test compounds were reconstituted in dimethyl sulphoxide (DMSO) to give a final concentration of 40 mM. Samples were stored at 4 °C until required. Cells were seeded and treated as described in 3.6.1 Cells were first fixed and permeabilized using the IntraPrep kit as per manufacturer’s instructions (Beckman Coulter). This kit allows for the immunological detection of intracellular antigens by creating apertures in the cell membrane without affecting the morphology of the cell. Cleaved caspase-8 (Asp 391) and cleaved caspase-3 (Asp 175) monoclonal antibodies (Cell Signaling Technology, Danvers, MA, USA) were used to determine the presence of activated caspase-8 and caspase-3, respectively. Cells were first blocked using PBS containing 0.5% BSA and thereafter incubated with the antibodies separately (1:200 for caspase-8 and 1:100 for caspase-3) for 1 h at 37 °C. The cells were washed and incubated with the Alexa 647 conjugated secondary antibody (1:1000) for 30 min at 37 °C in the dark. Both cell lines were incubated at 37 °C in a humidified 5% CO_2_ and then treated with the IC_50_ values of each compound for 24 h. 200 µL Aliquots of the diluted compound in fresh medium was used to treat cells after aspiration of seeding medium. Treatment medium was aspirated from all wells and replaced with 100 μL of Hoechst 33342 nuclear dye (5 μg/mL) and incubated for 10 min at RT.

#### 3.7.2. Data Quantification

Quantification of live and dead cells for the assay was performed using the ImageXpress Micro XLS Widefield Microscope and acquired images analysed using the MetaXpress software and Multi-Wavelength Cell Scoring Application Module and the Cell Cycle Module. Acquired data was transferred to an EXCEL spreadsheet and data was analysed and processed.

### 3.8. Tubulin Polymerization Assay

Tubulin polymerization assays were conducted using the tubulin polymerization assay kit following the manufacturer’s instructions (Cytoskeleton, Inc., Denver, CA, USA). Briefly, 50 μL of 1.3 mg/mL tubulin (>99% pure) proteins in G-PEM buffer (consisting of 80 mmol/L PIPES 2 mmol/L MgCl_2_, 0.5 mmol/L EGTA, 1 mmol/L GTP, and 15% glycerol at pH 6.9,) was placed in a quartz cuvette in the presence of the test agent (concentration: 0.25 µM). Polymerization was measured at every 3 s for 1 h using an Applied Photophysics Chirascan spectroflourimeter (excitation at 360 nm and emission at 420 nm) at 37 °C.

### 3.9. Methodology for Docking Studies

Docking of the compounds **2a**, **4a** and **5e**–**h** was performed to the crystal structure of a tubulin heterodimer (PDB code:1TUB) [29]. using the CDOCKER protocol [30] in Discovery Studio 2017. Prior to performing the docking, compounds were drawn using Discovery Studio and prepared using the ‘Prepare Ligand’ protocol. The protein structure was obtained from the Protein Data Bank, prepared using the ‘Prepare Protein’ protocol in Discovery Studio which included: removing any existing ligands bound to the model and binding sites defined from receptor cavities as well as all water molecules, protonate the protein at pH 7.5, The receptor cavity in this tubulin structure is present between the two monomers of the tubulin dimer structure. Docking was performed using CHARMm force field and the best conformation of the ligand selected and evaluated.

## 4. Conclusions

The observed increased cytotoxicity of compounds **5e**–**h** against the A549 and HeLa cell lines and their strong inhibitory effect against tubulin polymerization suggest that a combination of the strong hydrogen bonding 7-acetamido and the 3-trifluoroacetyl groups on the 2-aryl-5-bromoindole framework is desirable for biological activity. An acetamido group elicited some cytotoxicity in derivatives **4b**–**d** against both cell lines, which increased significantly upon incorporation of a trifluoroacetyl group in **5e**–**h**. This observation suggests the importance of a nitrogen-based substituent on the fused benzo ring of indole derivatives in analogy with the literature precedence on the increased cytotoxicity of analogues indoles against the HeLa and A549 cell lines as well as HIV-1 inhibition activity [6]. The lack of cytotoxicity for the C-3 unsubstituted 2-arylindoles **2a-d** or their 3-trifluoroacetyl derivatives **5a**–**d** in our view suggest that an acetyl group attached to the fused benzo ring of a 2-arylindole framework is not desirable for the cytotoxicity of polysubstituted indole derivatives. The immunochemical experiments, on the other hand, revealed that the 3-trifluoroacetyl derivatives **5e**–**h** may induce apoptosis in a caspase-dependent manner for both cell lines. Experimental and molecular docking of **5e**–**h** into the colchicine-binding site of tubulin suggest that these compounds have potential to inhibit tubulin polymerization to elicit anticancer activity. These results pave the way for the development of the 3-trifluoroacetyl substituted indole derivatives as anticancer agents.

## Figures and Tables

**Table 1:** Percentage yields of the compounds **5a**–**h** and the corresponding reaction intermediates **2**–**4**.

Ar	2	3	4	5a–d	5e–h
C_6_H_5_-	92 (**2a**)	90 (**3a**)	81 (**4a**)	92 (**5a**)	84 (**5e**)
4-FC_6_H_4_-	84 (**2b**)	87 (**3b**)	76 (**4b**)	84 (**5b**)	78 (**5f**)
3-ClC_6_H_4_-	85 (**2c**)	82 (**3c**)	73 (**4c**)	85 (**5c**)	80 (**5g**)
4-MeOC_6_H_4_-	77 (**2d**)	80 (**3d**)	76 (**4d**)	77 (**5d**)	52 (**5h**)

**Table 2:**
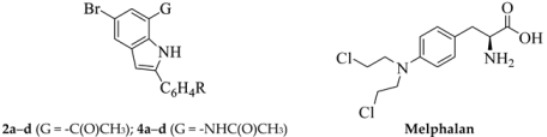
IC_50_ values of compounds **2a**–**d**, **4a**–**d** and Melphalan against the A549 and HeLa cells.

Compound	G	Ar	Cancer Cells IC_50_ (µM)	
			(A549)	HeLa
**2a**	-C(O)CH_3_	C_6_H_5_-	>200	>200
**2b**	-C(O)CH_3_	4-FC_6_H_4_-	>200	>200
**2c**	-C(O)CH_3_	3-ClC_6_H_4_-	>200	>200
**2d**	-C(O)CH_3_	4-MeOC_6_H_4_-	>200	>200
**4a**	-NHC(O)CH_3_	C_6_H_5_-	>200	>200
**4b**	-NHC(O)CH_3_	4-FC_6_H_4_-	116.2 ± 0.78	122.4 ± 1.02
**4c**	-NHC(O)CH_3_	3-ClC_6_H_4_-	154.6 ± 0.90	126.9 ± 1.00
**4d**	-NHC(O)CH_3_	4-MeOC_6_H_4_-	146.8 ± 1.00	125.0 ± 0.88
**Melphalan**	-	-	30.66 ± 0.76	37.16 ± 0.22

**Table 3:**
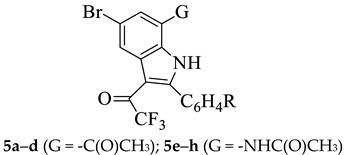
IC_50_ values of compounds **5a**–**h** against A549 and HeLa cells.

Compound	G	Ar	Cancer Cells IC_50_ (µM)	
			(A549)	HeLa
**5a**	-C(O)CH_3_	C_6_H_5_-	>200	>200
**5b**	-C(O)CH_3_	4-FC_6_H_4_-	>200	>200
**5c**	-C(O)CH_3_	3-ClC_6_H_4_-	>200	>200
**5d**	-C(O)CH_3_	4-MeOC_6_H_4_-	>200	>200
**5e**	-NHC(O)CH_3_	C_6_H_5_-	9.94 ± 0.99	12.89 ± 1.11
**5f**	-NHC(O)CH_3_	4-FC_6_H_4_-	5.03 ± 0.71	7.95 ± 0.90
**5g**	-NHC(O)CH_3_	3-ClC_6_H_4_-	2.72 ± 0.43	8.74 ± 0.94
**5h**	-NHC(O)CH_3_	4-MeOC_6_H_4_-	3.26 ± 0.51	10.72 ± 1.03
**Melphalan**	-		30.66 ± 0.76	37.16 ± 0.22

**Table 4:** IC_50_ values of **5e**–**h** against tubulin using indol-3-carbinol and colchicine as positive controls.

Compound	IC_50_ (µM)	S.D.
**5e**	3.18 × 10^−5^	±0.06
**5f**	3.28 × 10^−7^	±0.66
**5g**	2.42 × 10^−5^	±0.01
**5h**	4.86 × 10^−6^	±0.08
**Indole-3-carbinol**	9.76 × 10^−5^	±0.27
**Colchicine**	9.88 × 10^−5^	±0.17

## Supplementary Materials

The following materials are available online at http://www.mdpi.com/1424-8247/11/2/59/s1. Copies of ^1^H- & ^13^C-NMR spectra for compounds **2a**–**d**, **3a**–**d**, **4a**–**d** and **5a**–**h** (Figure S1), % cell viability for compounds of **2a**–**d**, **4a**–**d** and **5a**–**h** (Figure S2), dose response curves for compounds **5e**–**h** (Figure S3) and spread sheet for statistical analysis which contains p values for each test (Figure S4).

## References

[1] Lu Y., Chen J., Xiao M., Li W., Miller D.D. (2012). An overview of tubulin inhibitors that interact with the colchicine binding site. Pharm. Res..

[2] Jordan M.A. (2002). Anti-cancer agents. Curr. Med. Chem..

[3] Gastpar R., Goldbrunner M., Marko D., Von Angerer E. (1998). Methoxy-substituted 3-formyl-2-phenylindoles inhibit tubulin polymerization. J. Med. Chem..

[4] Dong M., Liu F., Zhou H., Zhai S., Yan B. (2016). Novel natural product- and privileged scaffold-based tubulin inhibitors targeting the colchicine binding site. Molecules.

[5] Kaur R., Kaur G., Gill R.K., Soni R., Bariwal J. (2014). Recent developments in tubulin polymerization inhibitors: An overview. Eur. J. Med. Chem..

[6] Hu H., Wu J., Ao M., Wang H., Zhou T., Xue Y., Qiu Y., Fang M., Wu Z. (2016). Synthesis, structure–activity relationship studies and biological evaluation of novel 2,5-disubstituted indole derivatives as anticancer agents. Chem. Biol. Drugs Des..

[7] Cooper L.C., Chicchi G.G., Dinnell K., Elliott J.M., Hollingworth G.J., Kurtz M.M., Locker K.L., Morrison D., Shaw D.E., Tsao K.-L. (2001). 2-Aryl indole NK1 receptor antagonists: Optimisation of indole substitution. Bioorg. Med. Chem. Lett..

[8] Sisa M., Pla D., Altuna M., Francesch A., Cuevas C., Albericio F., Alvarez M. (2009). Total synthesis and antiproliferative activity screening of (±)-Aplicyanins A, B and E and related analogues. J. Med. Chem..

[9] Watterson S.H., Dhar T.G.M., Ballentine S.K., Shen Z., Barrish J.C., Cheney D., Fleener C.A., Roleau K.A., Townsend R., Hollenbaugh D.L. (2003). Novel indole-based inhibitors of IMPDH: Introduction of hydrogen bond acceptors at indole C-3. Bioorg. Med. Chem. Lett..

[10] Thomas J.B., Giddings A.M., Wiethe R.W., Olepu S., Warner K.R., Sarret P., Gendron L., Longpre J.-M., Zhang Y., Runyon S.P. (2014). Identification of *N*-[(5-{[(4-methylphenyl)sulfonyl]amino}-3-(trifluoroacetyl)-1*H*-indol-1-yl)acetyl]-L-leucine (NTRC-824), a neurotensin-like nonpeptide compound selective for the neurotensin receptor type 2. J. Med. Chem..

[11] Usachev B.I. (2016). 1-/2-/3-Fluoroalkyl-substituted indoles, promising medicinally and biologically beneficial compounds: Synthetic routes, significance and potential applications. J. Fluor. Chem..

[12] Cade H.C., Blocker M., Shaik A. (2015). Synthesis of indole-derived fluorine-containing amino acids. J. Nat. Sci..

[13] Lo’pez S.E., Salazar J. (2013). Trifluoroacetic acid: Uses and recent applications in organic synthesis. J. Fluor. Chem..

[14] Mphahlele M.J., Mmonwa M.M., Makhafola T.J. (2016). In vitro cytotoxicity of novel 2,5,7-tricarbo-substituted indoles derived from 2-amino-5-bromo-3-iodoacetophenone. Bioorg. Med. Chem..

[15] Choi H.-S., Cho M.-C., Lere H.G., Yoon D.-Y. (2010). Indole-3-carbinol induces apoptosis through p53 and activation of caspase-8 pathway in lung cancer A549 cells. Food Chem. Toxicol..

[16] Mphahlele M.J., Maluleka M.M. (2016). Trifluoroacetylation of indole-chalcones derived from the 2-amino-3- (arylethynyl)-5-bromo-iodochalcones. J. Fluor. Chem..

[17] Mphahlele M.J., Mmonwa M.M., Choong Y.S. (2017). Synthesis and evaluation of *N*-(3-trifluoroacetyl-indol-7-yl)acetamides for potential in vitro antiplasmodial properties. Molecules.

[18] Yao S.-J., Ren Z.-H., Wang Y.-Y., Guan Z.-H. (2016). Friedel–Crafts fluoroacetylation of indoles with fluorinated acetic acids for the synthesis of fluoromethyl indol-3-yl ketones under catalyst- and additive-free conditions. J. Org. Chem..

[19] Owa T., Yokoi A., Yamazaki K., Yoshimatsu K., Yamori T., Nagasu T. (2002). Array-based structure and gene expression relationship study of antitumor sulfonamides including *N*-[2-[(4-hydroxyphenyl)amino]-3- pyridinyl]-4-methoxybenzenesulfonamide and *N*-(3-chloro-7-indolyl)-1,4-benzenedisulfonamide. J. Med. Chem..

[20] Kroemer G., Galluzzi L., Vandenabeele P., Abrams J., Alnemri E.S., Baehrecke E.H., Blagosklonny M.V., El-Deiry W.S., Golstein P., Green D.R. (2009). Classification of cell death: Recommendations of the Nomenclature Committee on Cell Death 2009. Cell Death Differ..

[21] Fischer U., Janicke R.U., Schulze-Osthoff K. (2003). Many cuts to ruin: A comprehensive update of caspase substrates. Cell Death Differ..

[22] Fulda S., Debatin K.-A. (2006). Extrinsic versus intrinsic apoptosis pathways in anticancer chemotherapy. Oncogene.

[23] Taylor R.C., Cullen S.P., Martin S.J. (2008). Apoptosis: Controlled demolition at the cellular level. Nat. Rev. Mol. Cell Biol..

[24] Cohen G.M. (1997). Caspases; the executioners of apoptosis. Biochem. J..

[25] Kool E.T. (2001). Hydrogen bonding, base stacking, and steric effects in DNA replication. Annu. Rev. Biophys. Biomol. Struct..

[26] Kumar S., Mehndiratta S., Nepali K., Gupta M.K., Koul S., Sharma P.R., Saxena A.K., Dhar K.L. (2013). Novel indole-bearing combretastatin analogues as tubulin polymerization inhibitors. Org. Med. Chem. Lett..

[27] Hadzi D., Kidric J., Koller J., Mavri J. (1990). The role of hydrogen bonding in drug-receptor interactions. J. Mol. Struct..

[28] Farce A., Loge C., Gallet S., Lebegue N., Carato P., Chavatte P., Berthelot P., Lesieur D. (2004). Docking study of ligands into colchicine binding site of tubulin. J. Enzym. Inhib. Med. Chem..

[29] Nogales E., Wolf S.G., Downing K.H. (1998). Structure of the β-tubulin dimer by electron crystallography. Nature.

[30] Wu G., Robertson D.H., Brooks C.L., Vieth M. (2003). Detailed analysis of grid-based molecular docking: A case study of CDOCKER-A CHARMm-based MD docking algorithm. J. Comput. Chem..

