# Supplementary material for: Synthesis, Evaluation of Cytotoxicity and Molecular Docking Studies of the 7-Acetamido Substituted 2-Aryl-5-bromo-3-trifluoroacetylindoles as Potential Inhibitors of Tubulin Polymerization
Source: Pharmaceuticals (Basel). 2018 Jun 11;11(2):59. doi: 10.3390/ph11020059 (PMC6027433; doi:10.3390/ph11020059)
Supplement: Supplementary file 1 [file pharmaceuticals-11-00059-s001.pdf]

Supplementary data

Synthesis, evaluation of cytotoxicity and molecular docking studies of the 7-acetamide–substituted 2-aryl-5-bromo-3-trifluoroacetylindoles as potential inhibitors of tubulin polymerisation

M.J. Mphahlele, N. Parbhoo

Figure S1: ^{1}H- and ^{13}C-NMR spectra of compounds **2a–d**, **3a–d**, **4a–d** and **5a–h**
Figure S2: Cytotoxicity of compounds **2a–d**, **4a–d** and **5a–h** against A549 and HeLa cells
Figure S3: Dose response curves for **5e–h** against the A549 and HeLa cells
Figure S4: Spread sheet for statistical analysis which contains p values for each test
Figure S5: Excel spreadsheets for raw data and SD values for compounds **5e–h** as well as GraphPad Prism files for IC_{50} determination

Figure S1: ^{1}H- and ^{13}C-NMR spectra of compounds **2a–d**, **3a–d**, **4a–d** and **5a–h**

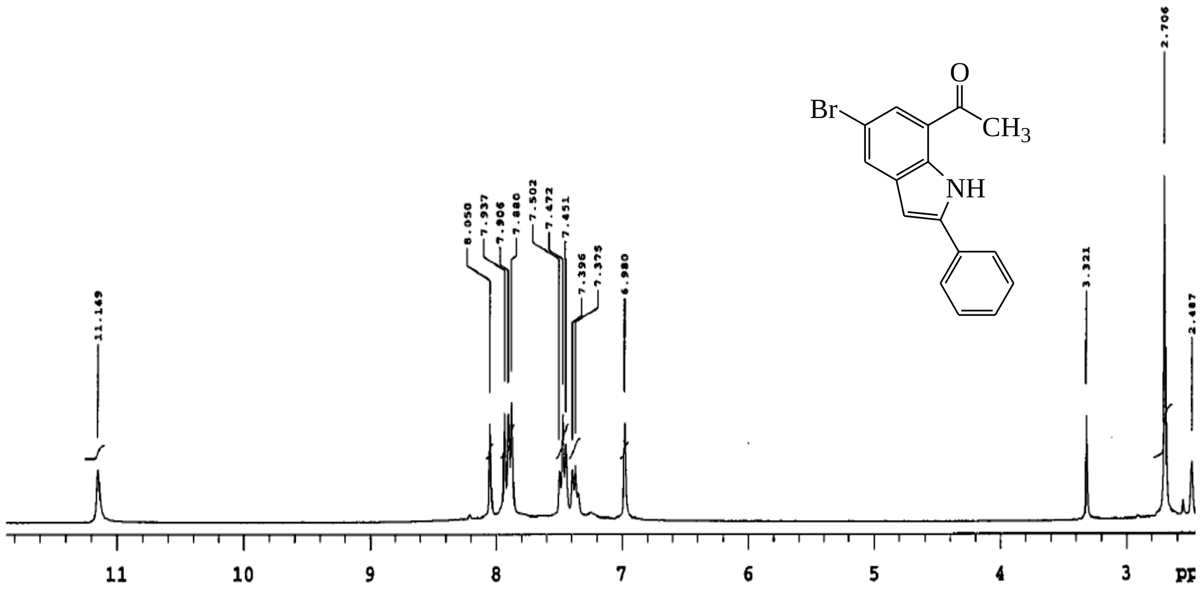

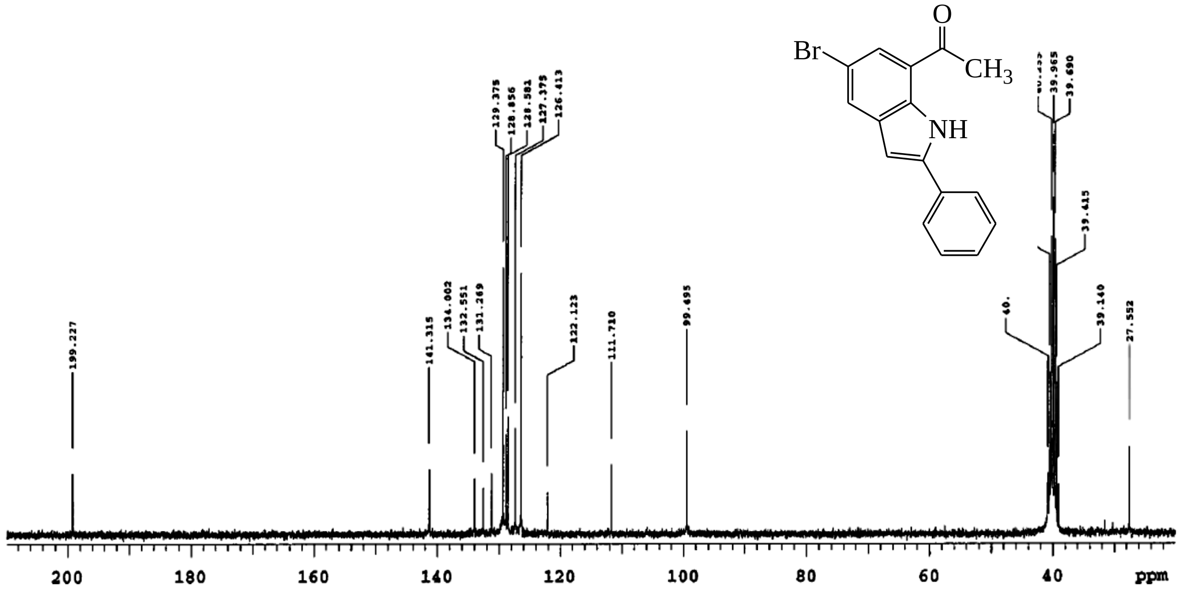

Figure S1.1: ^{1}H- and ^{13}C-NMR spectra of **2a** in DMSO-d_6 at 300 MHz and 75 MHz, respectively.

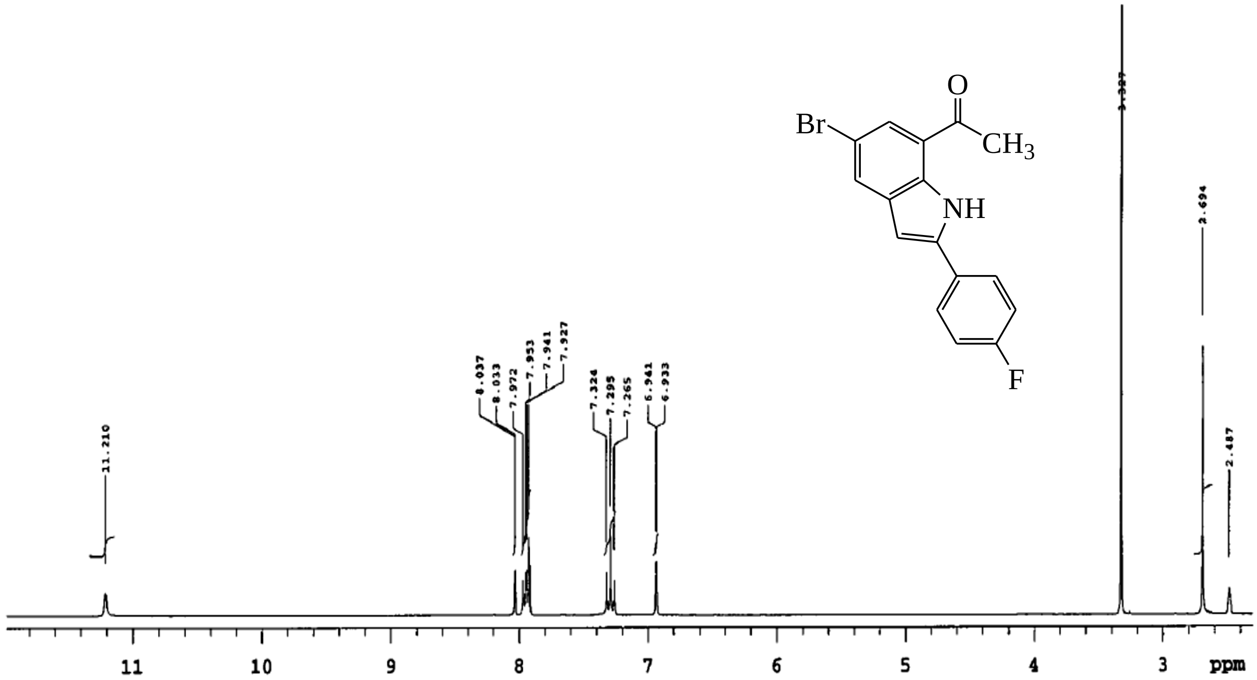

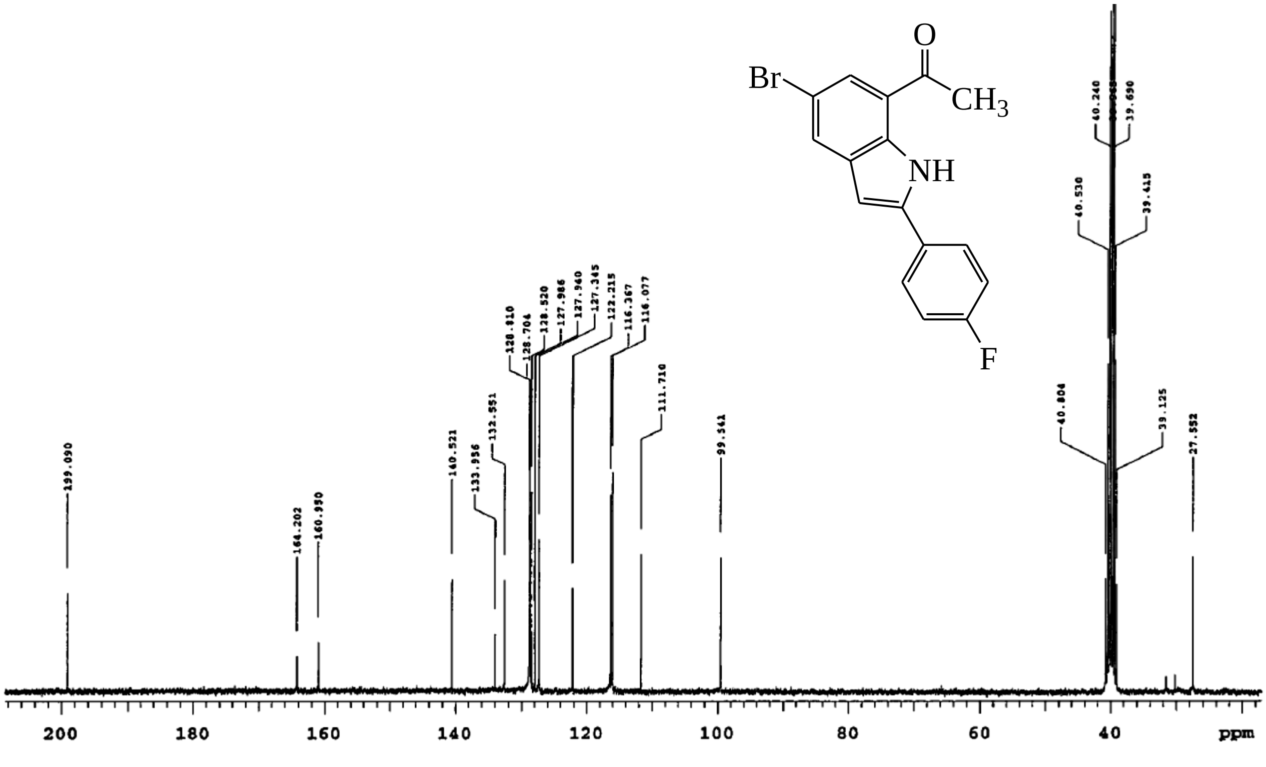

Figure S1.2: ^{1}H- and ^{13}C-NMR spectra of **2b** in DMSO-d_6 at 300 MHz and 75 MHz, respectively.

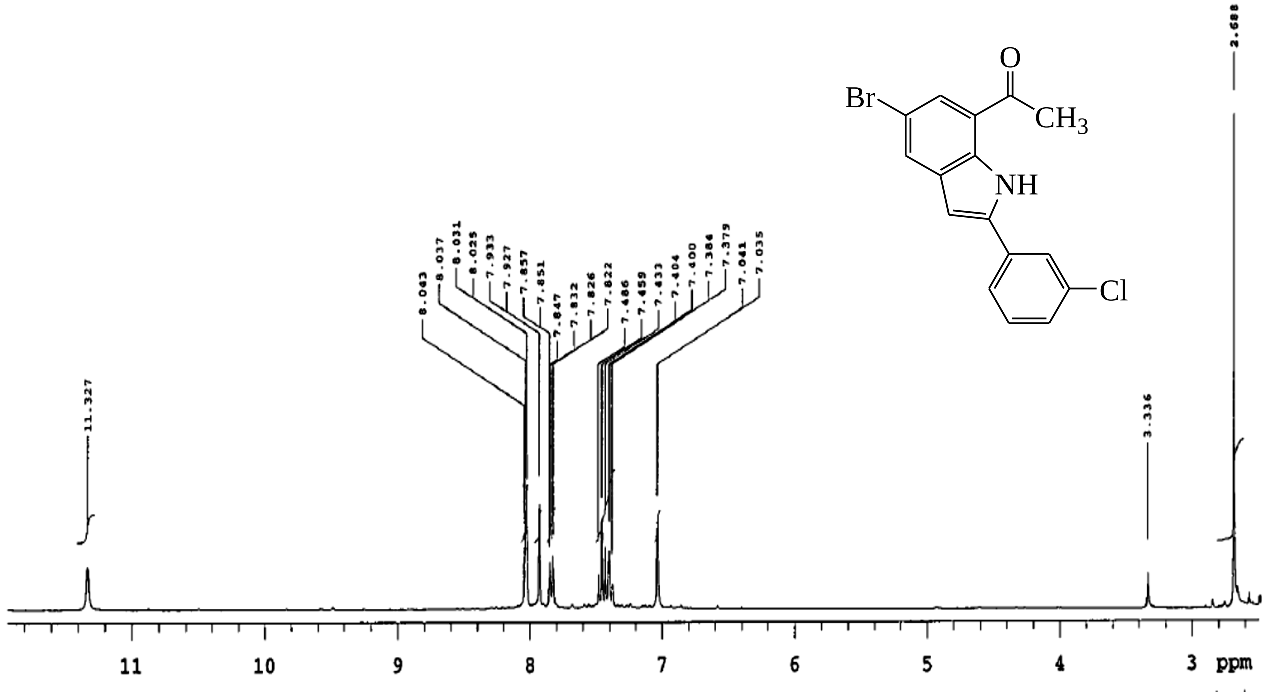

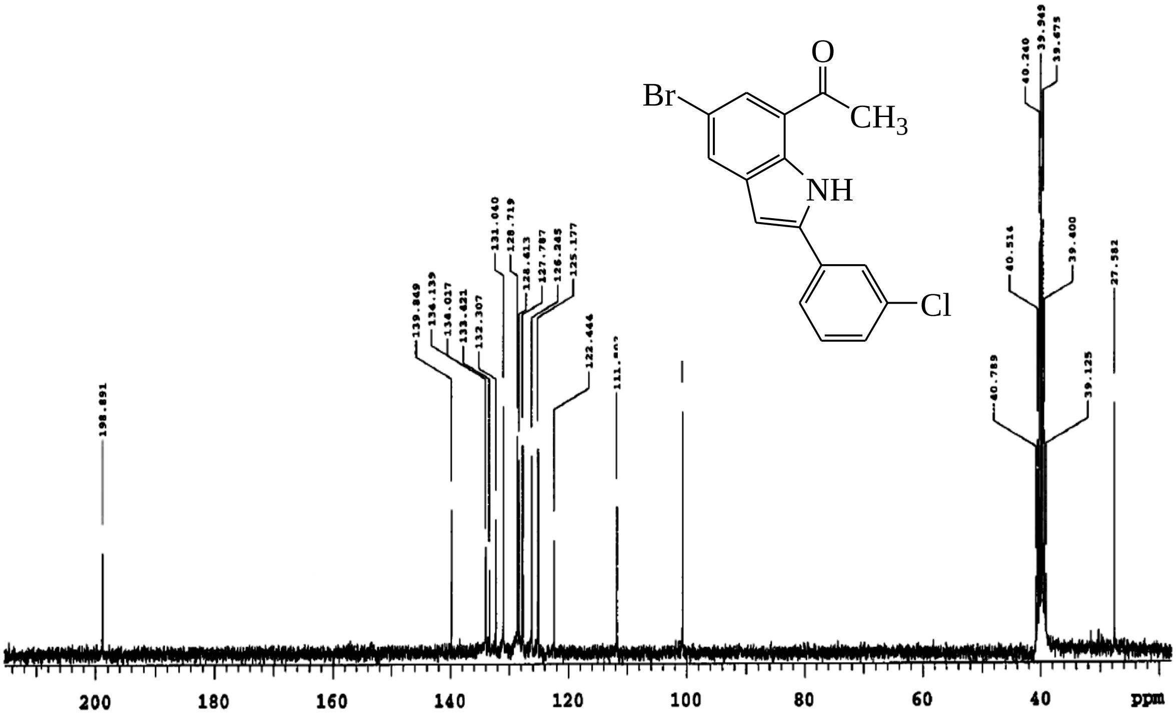

Figure S1.3: ^{1}H- and ^{13}C-NMR spectra of **2c** in DMSO-d_6 at 300 MHz and 75 MHz, respectively.

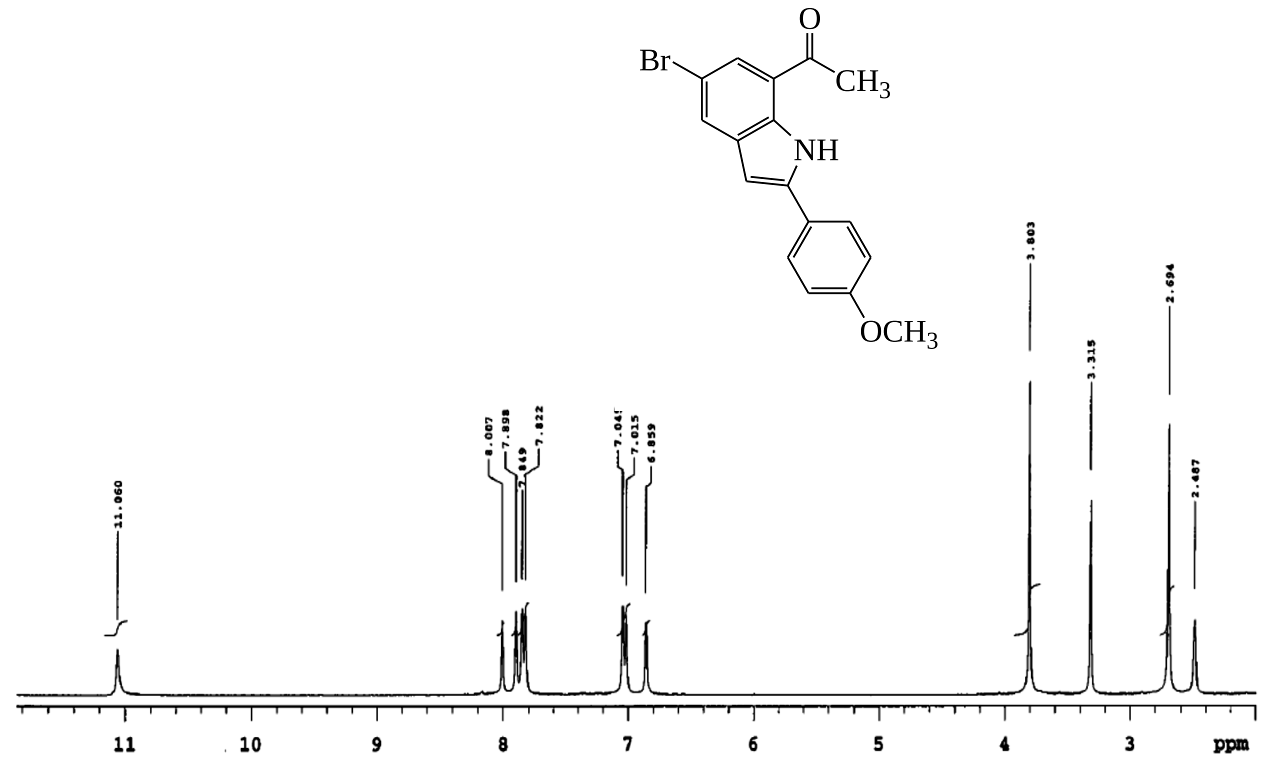

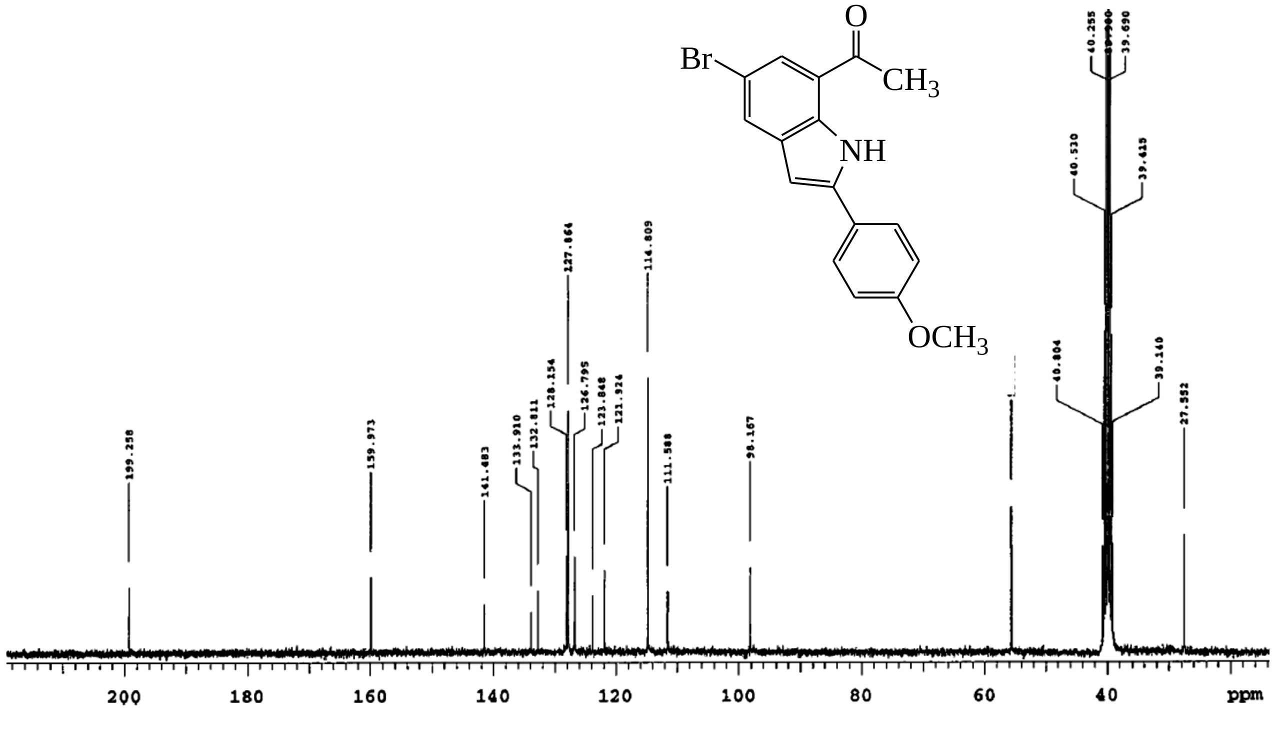

Figure S1.4: ^{1}H- and ^{13}C-NMR spectra of **2d** in DMSO-*d*$_6$ at 300 MHz and 75 MHz, respectively.

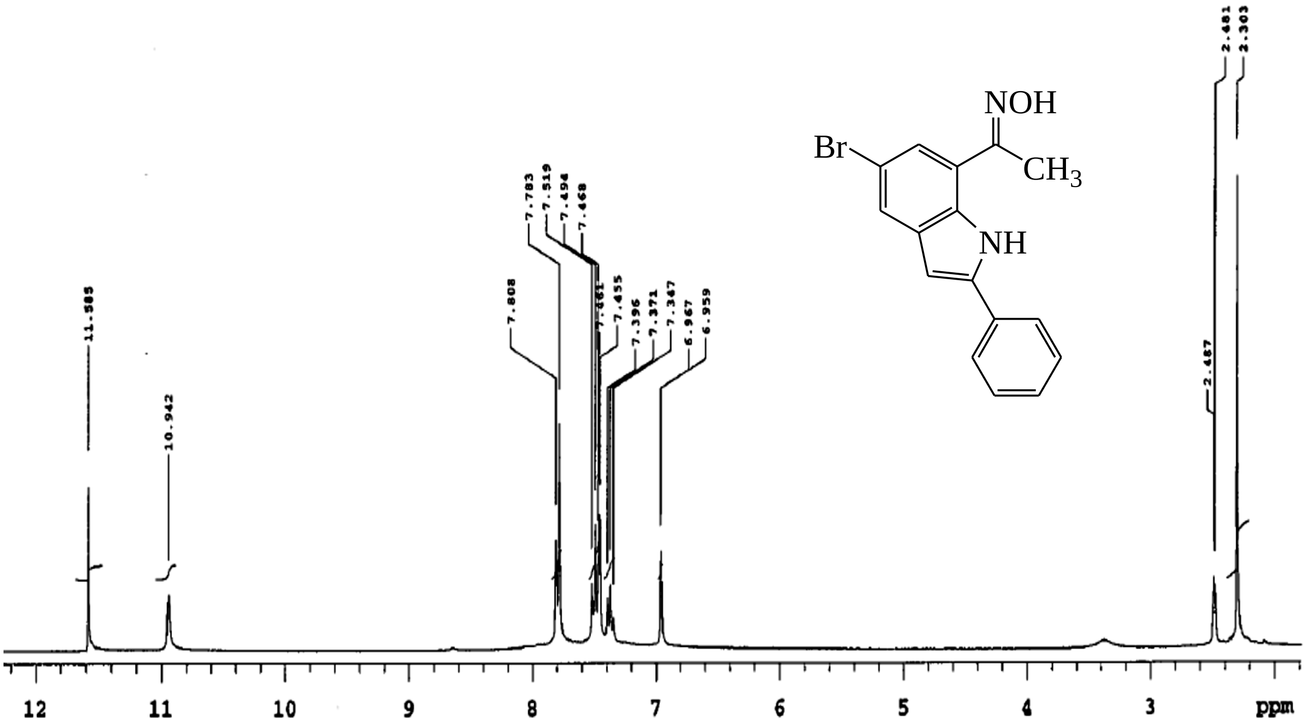

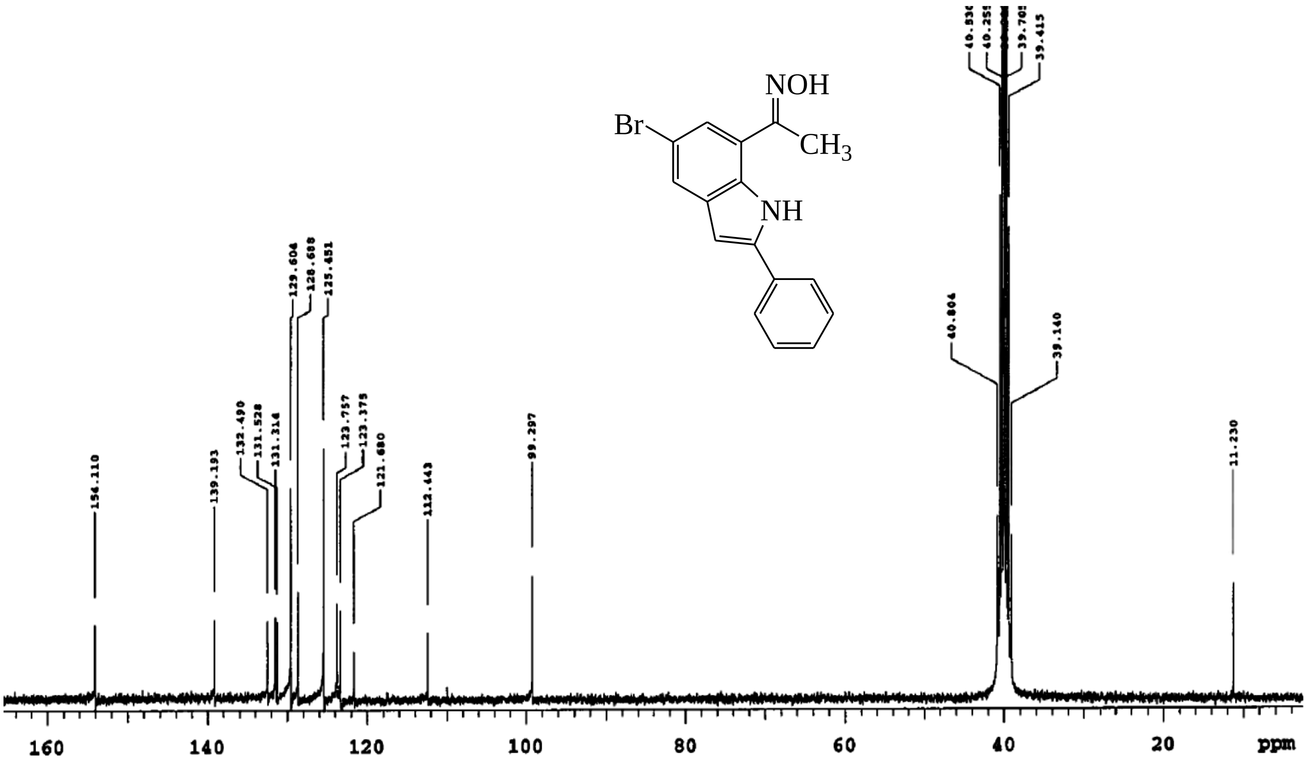

Figure S1.5: ^{1}H- and ^{13}C-NMR spectra of **3a** in DMSO-d_6 at 300 MHz and 75 MHz, respectively.

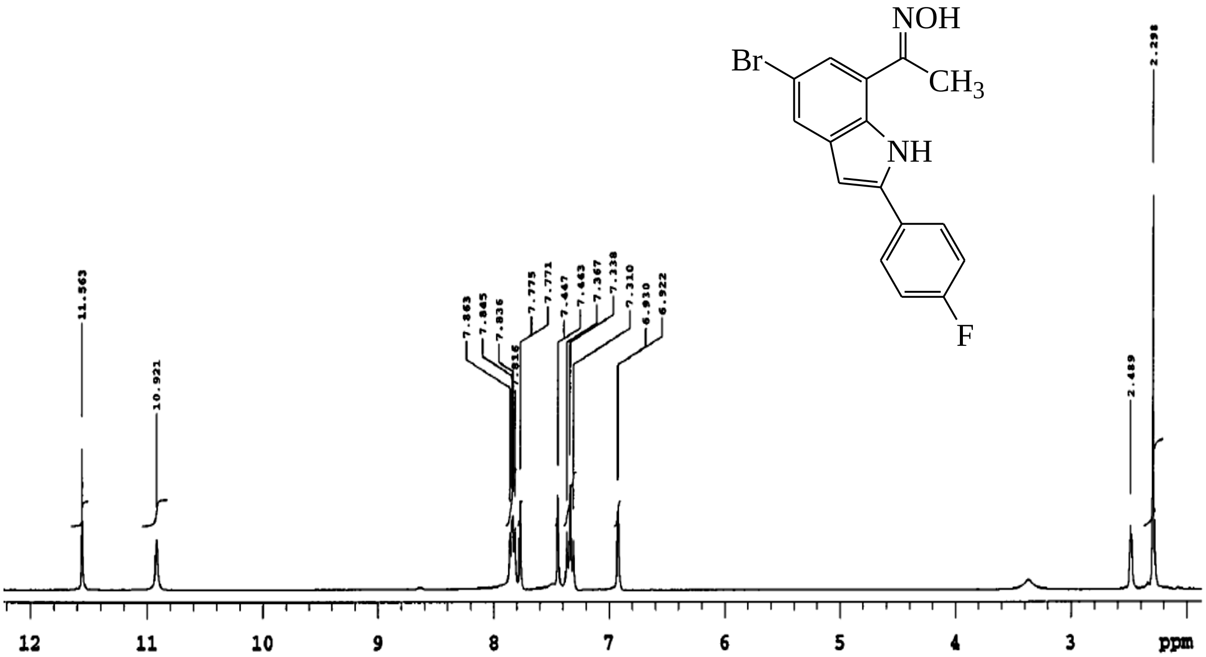

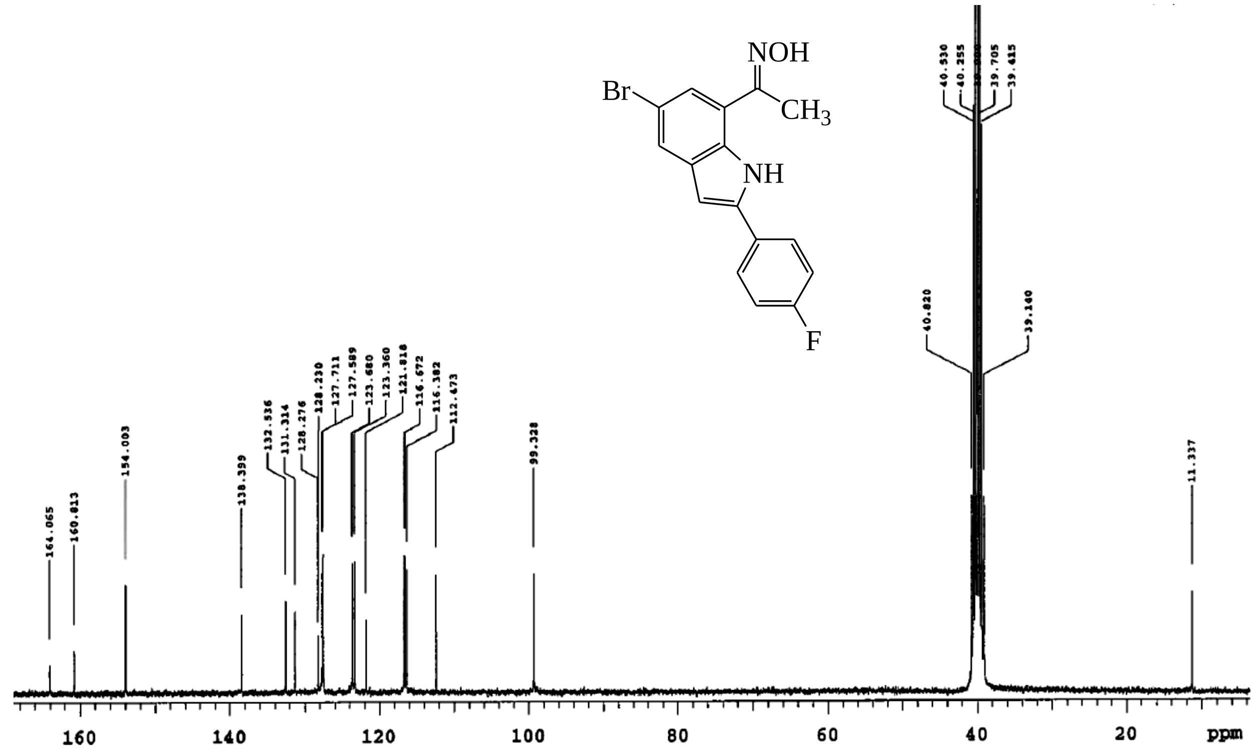

Figure S1.6: ^{1}H- and ^{13}C-NMR spectra of **3b** in DMSO-*d*$_6$ at 300 MHz and 75 MHz, respectively.

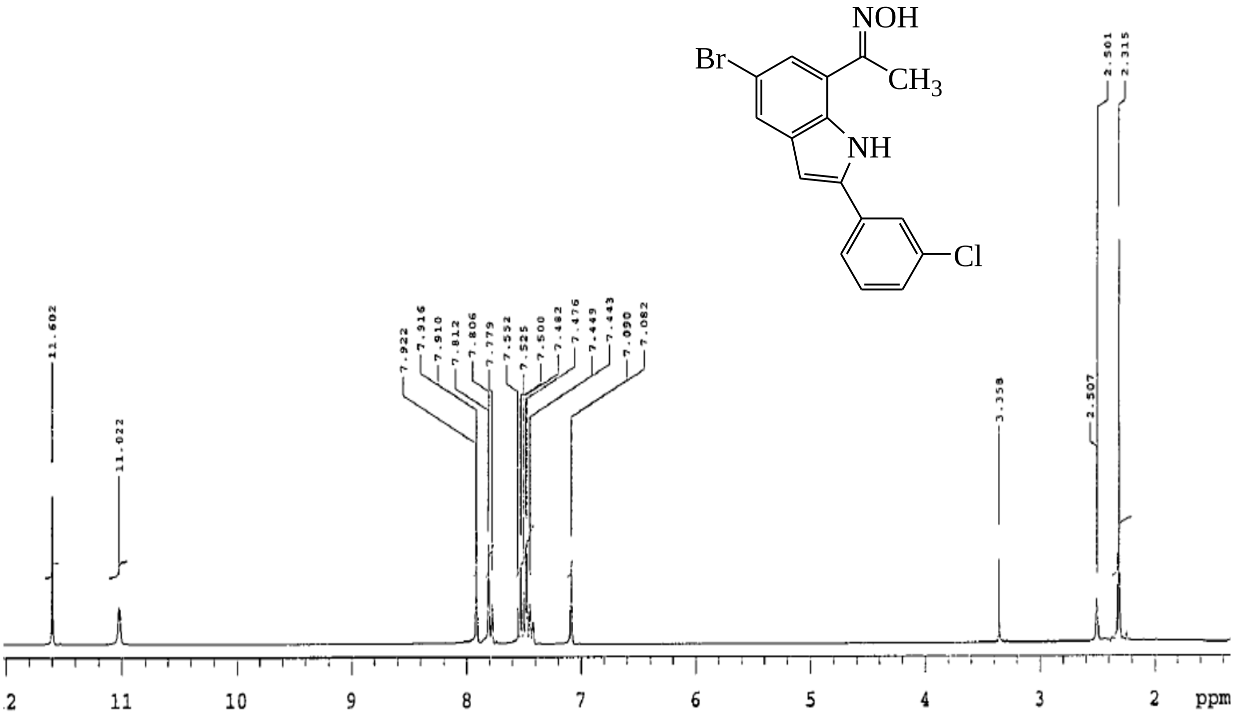

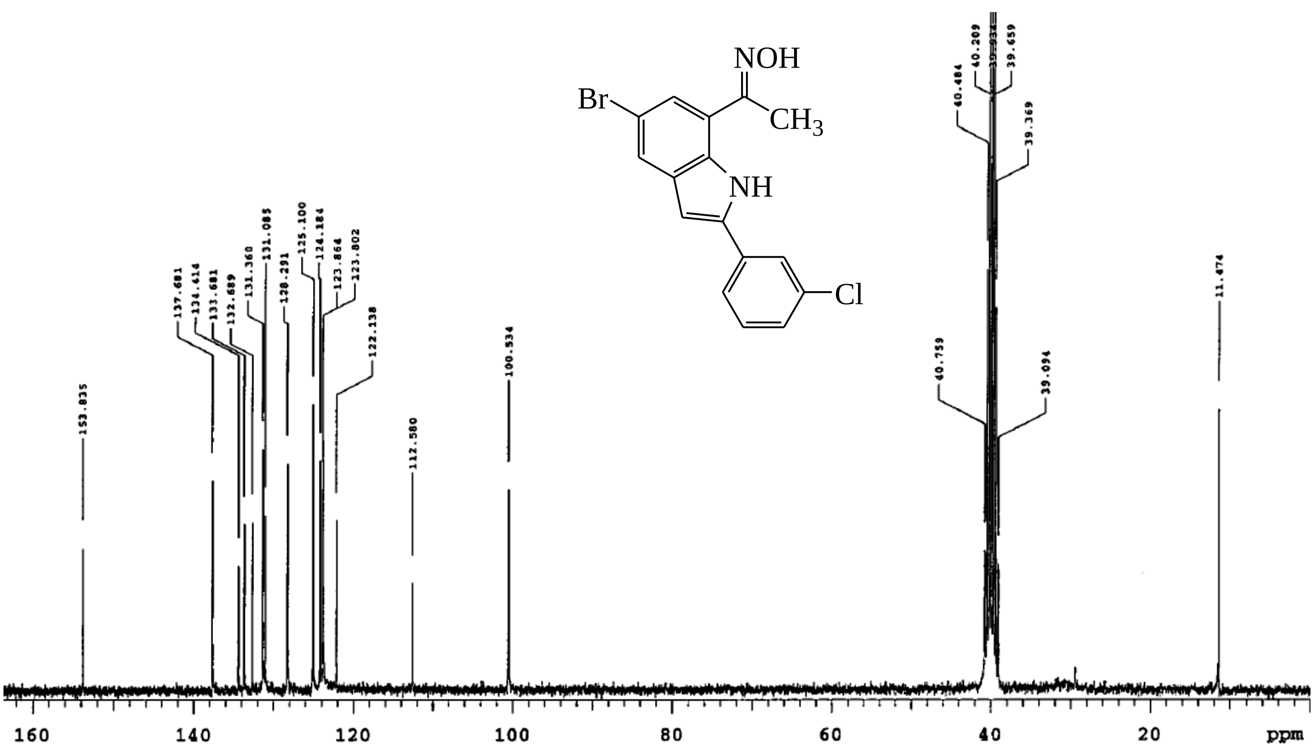

Figure S1.7: ^{1}H- and ^{13}C-NMR spectra of **3c** in DMSO-d_6 at 300 MHz and 75 MHz, respectively.

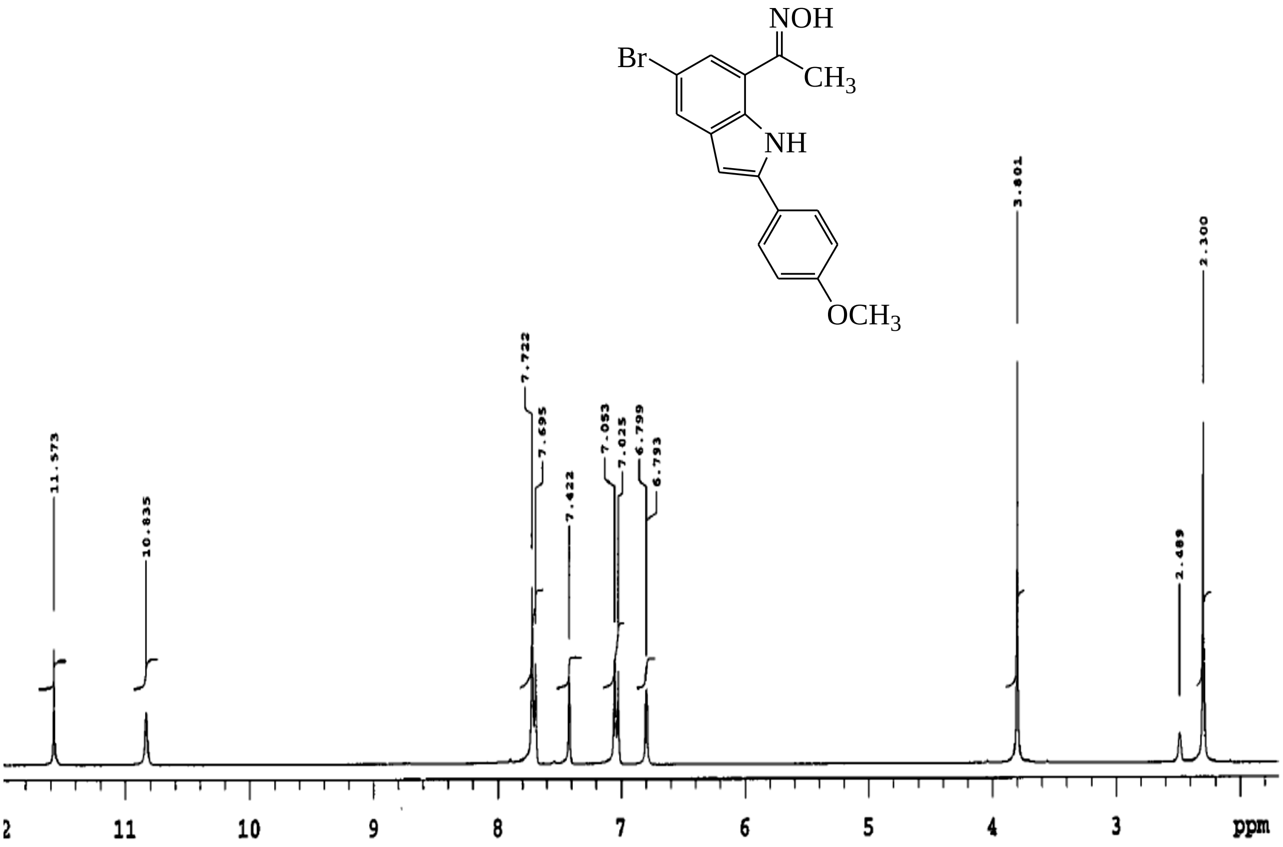

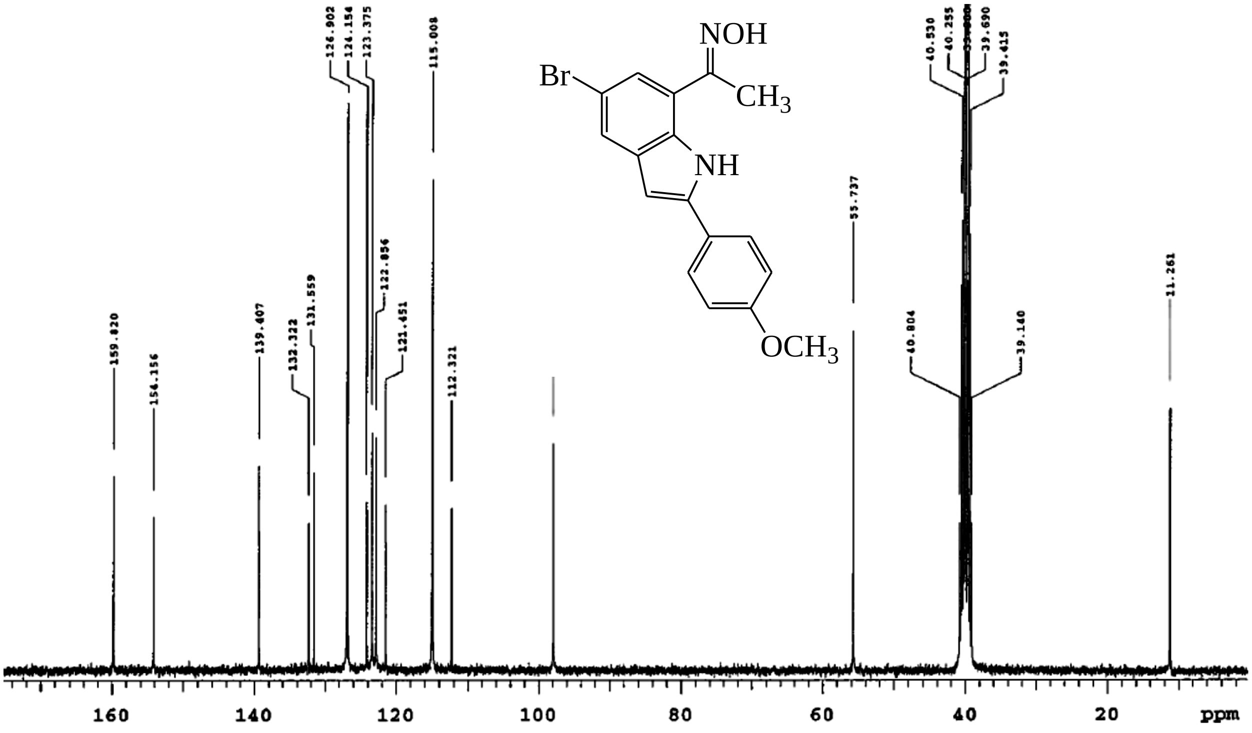

Figure S1.8: ^{1}H- and ^{13}C-NMR spectra of **3d** in DMSO-d_6 at 300 MHz and 75 MHz, respectively.

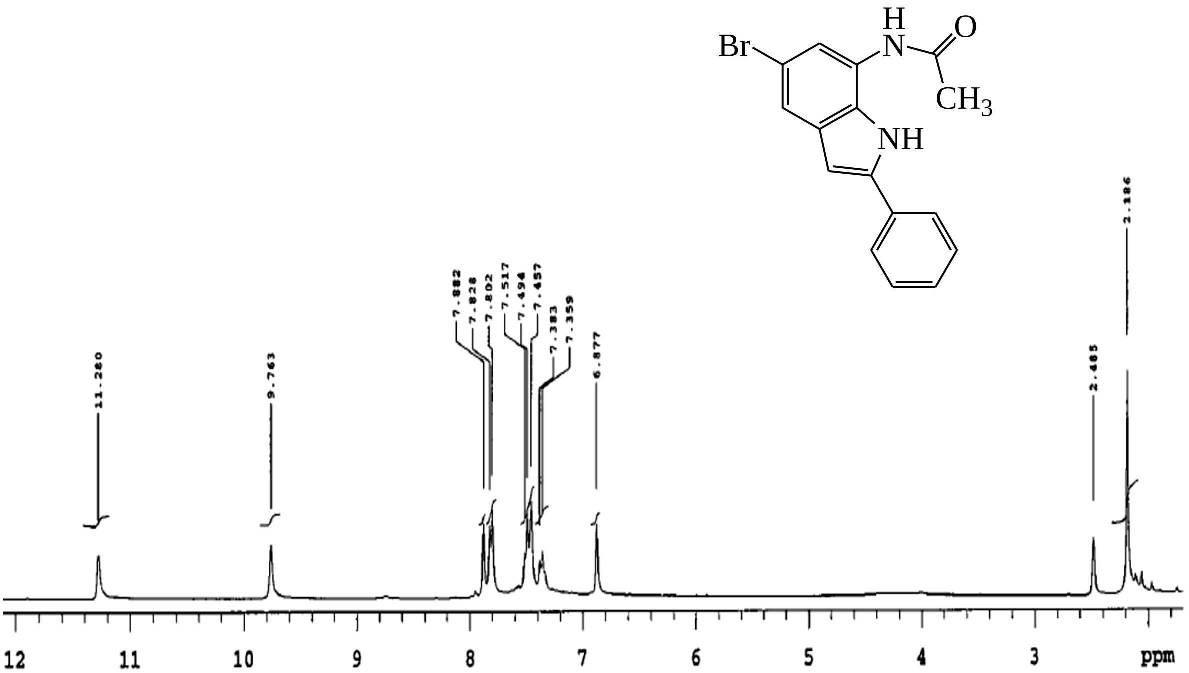

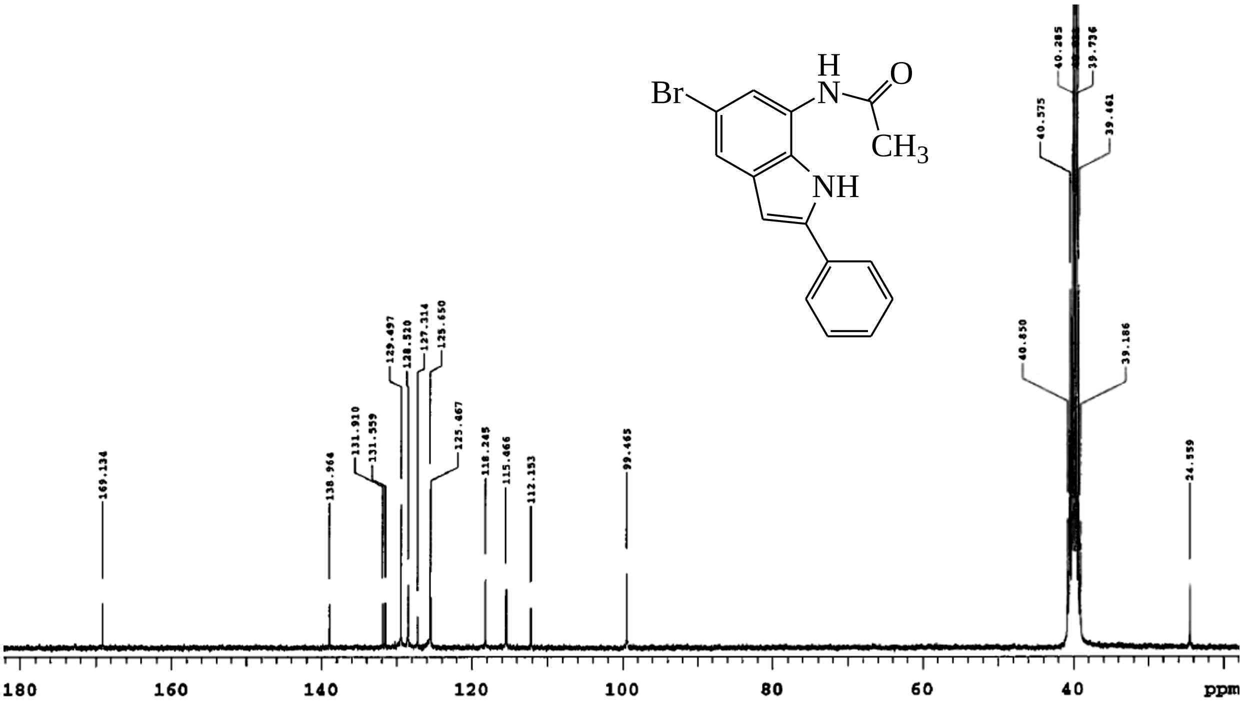

Figure S1.9: ^{1}H- and ^{13}C-NMR spectra of **4a** in DMSO-d_6 at 300 MHz and 75 MHz, respectively.

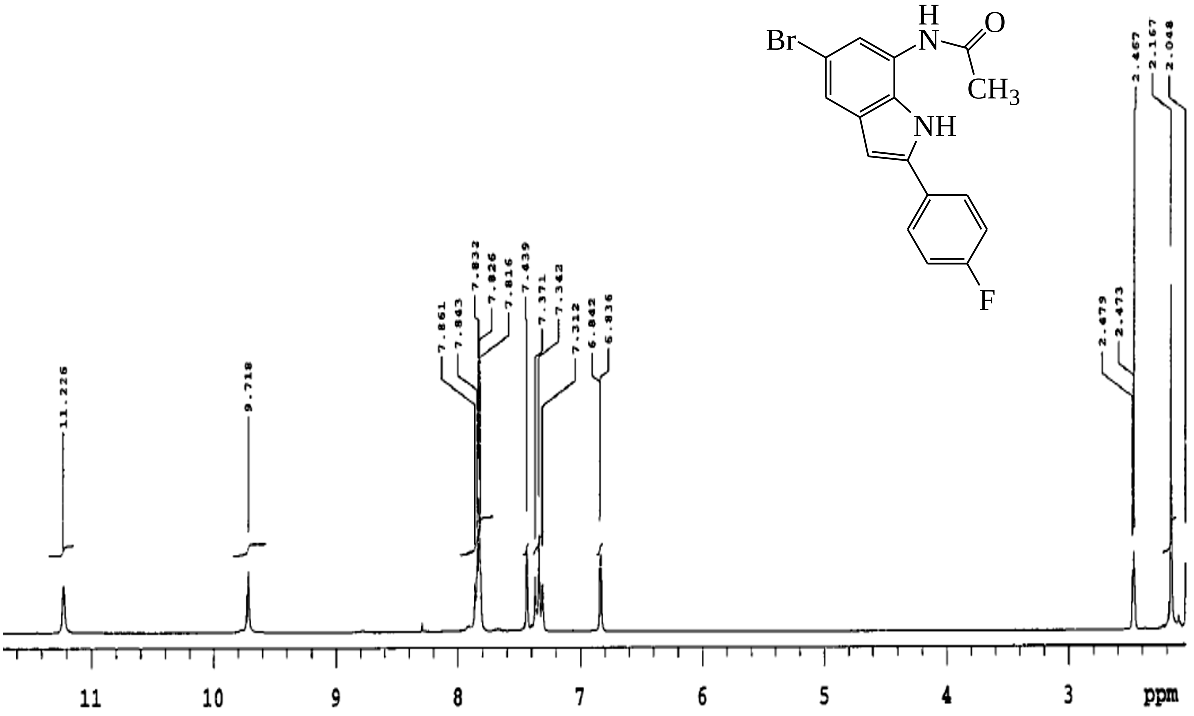

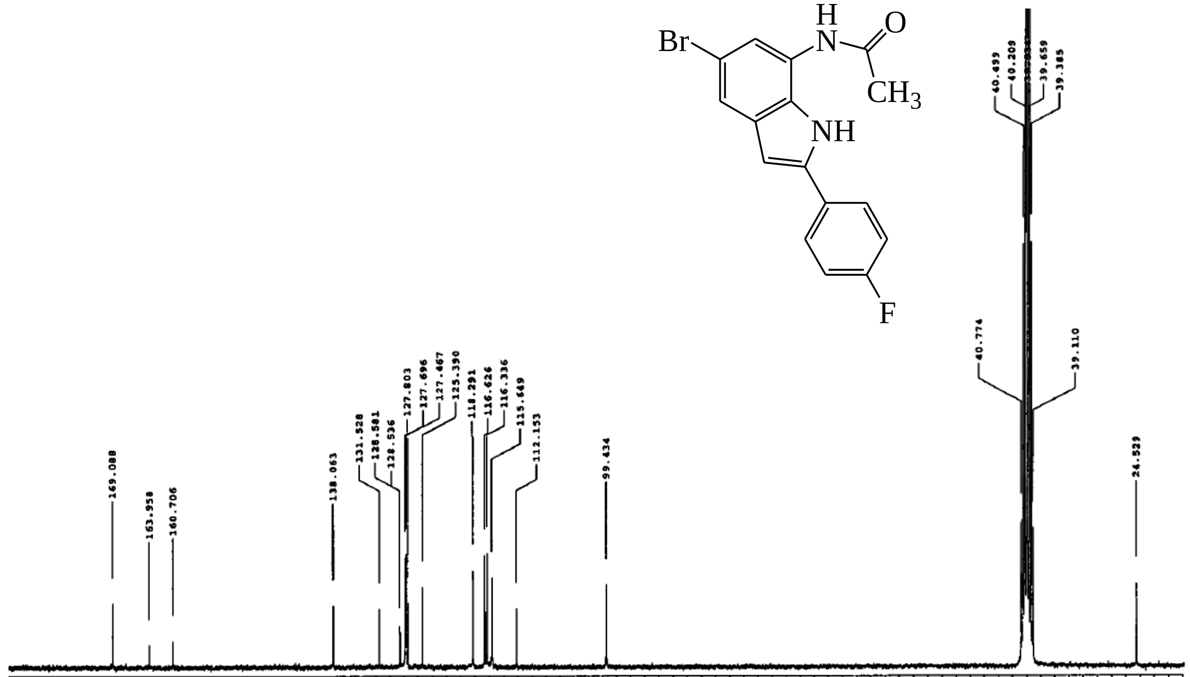

Figure S1.10: ^{1}H- and ^{13}C-NMR spectra of **4b** in DMSO-d_6 at 300 MHz and 75 MHz, respectively.

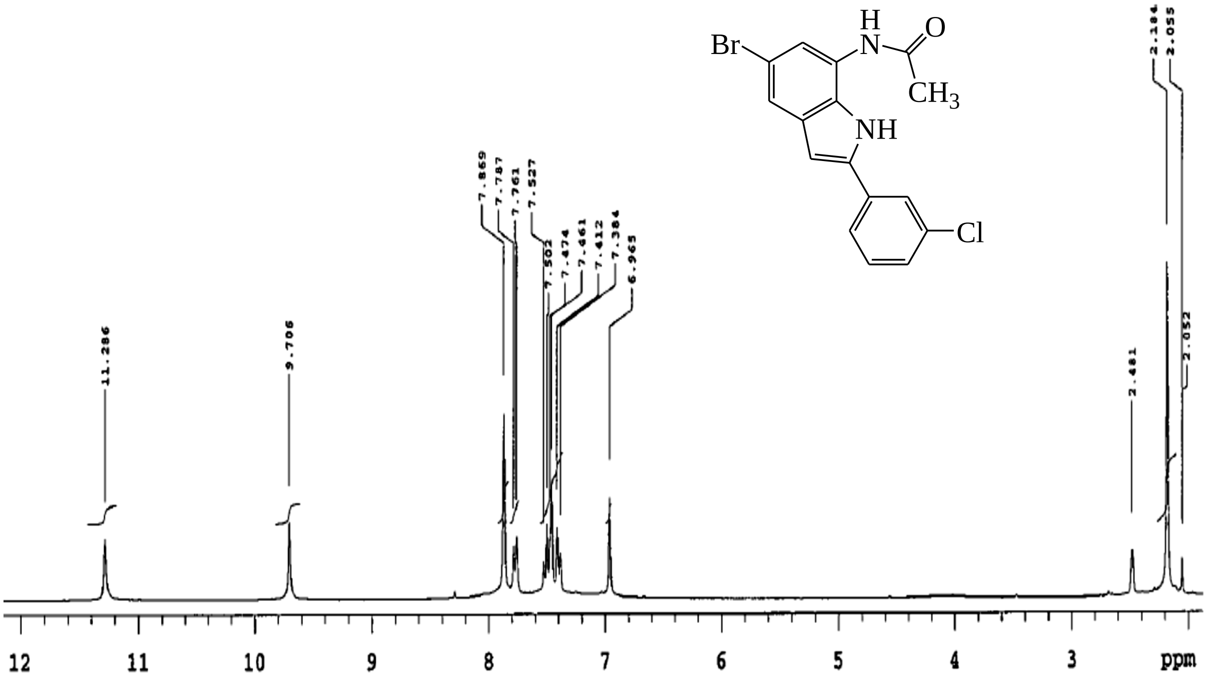

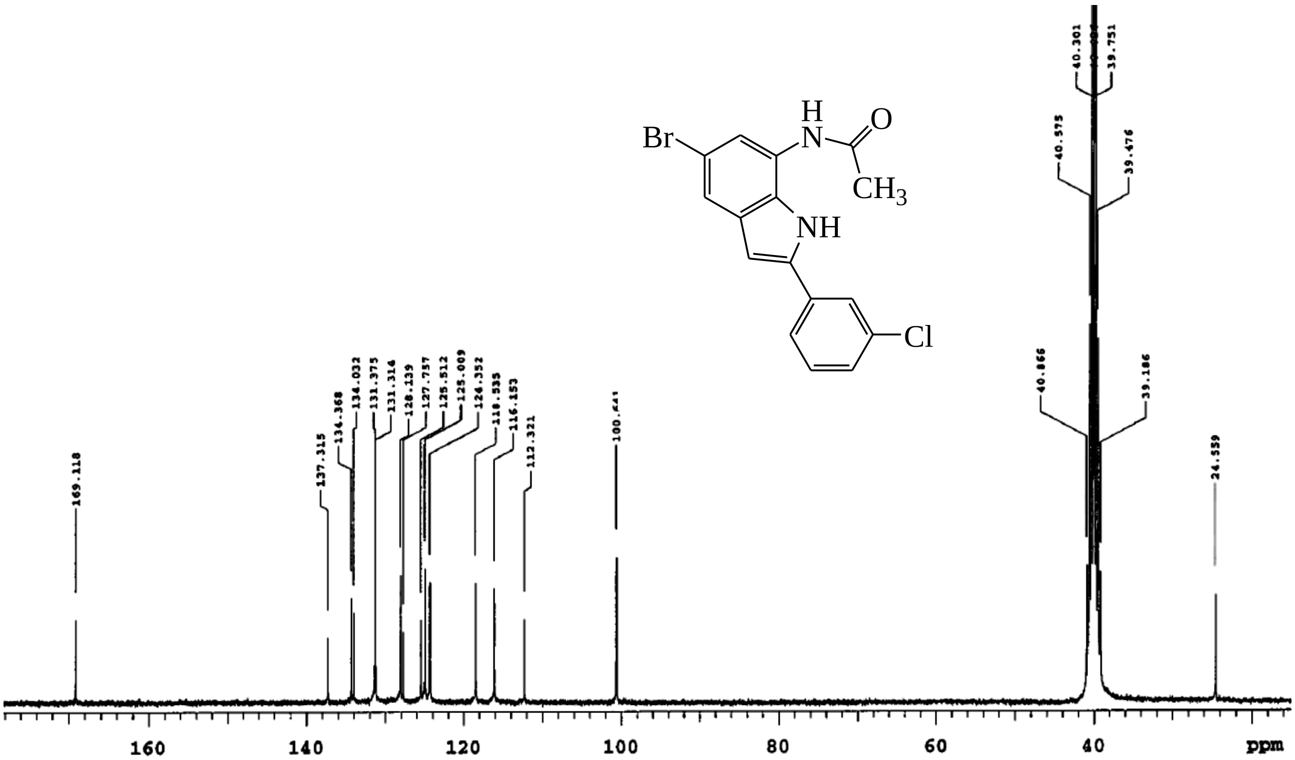

Figure S1.11: ^{1}H- and ^{13}C-NMR spectra of **4c** in DMSO-*d*$_6$ at 300 MHz and 75 MHz, respectively.

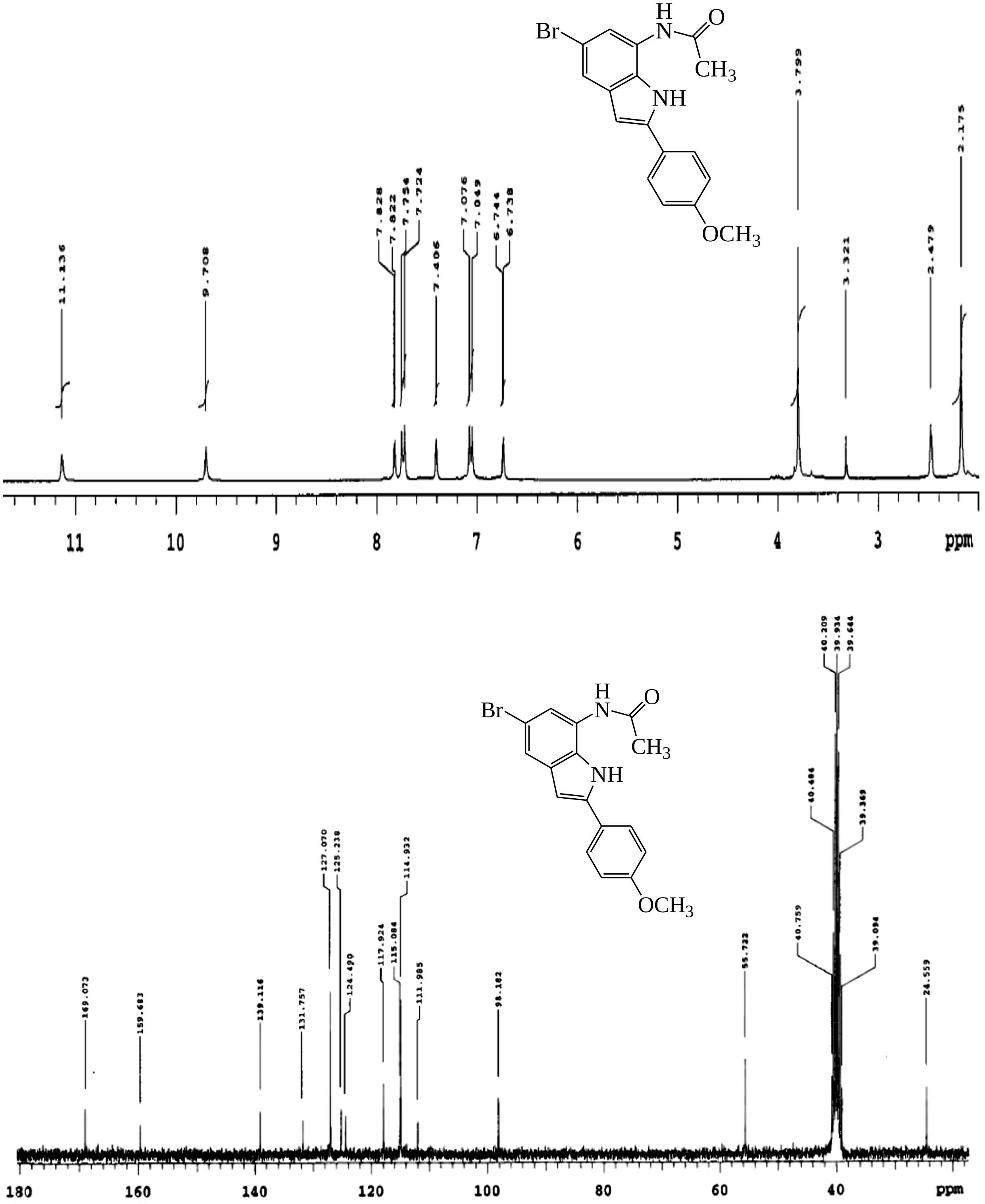

Figure S1.12: ^{1}H- and ^{13}C-NMR spectra of **4d** in DMSO-d_6 at 300 MHz and 75 MHz, respectively.

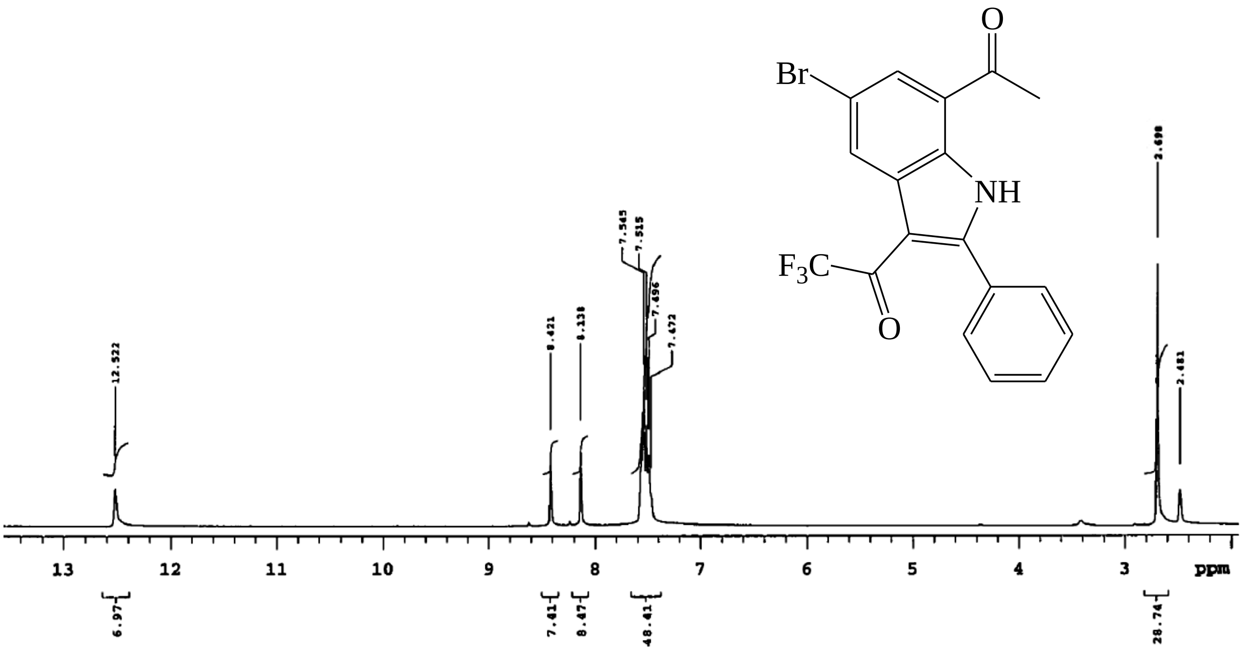

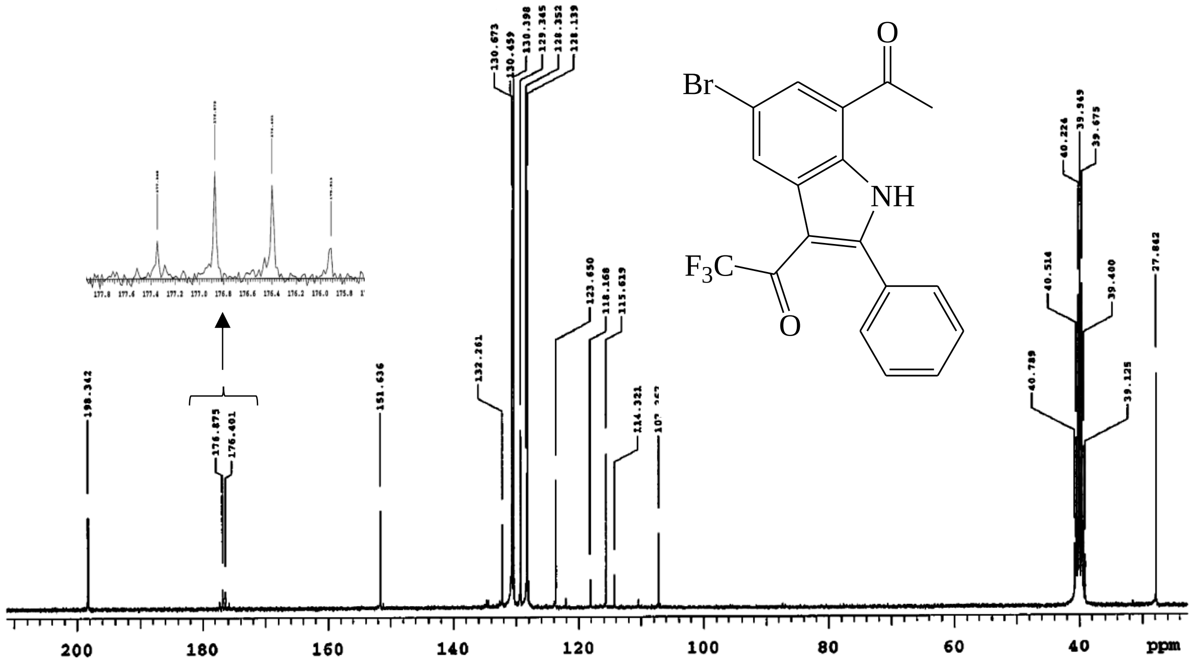

Figure S1.13: ^{1}H- and ^{13}C-NMR spectra of **5a** in DMSO-d_6 at 300 MHz and 75 MHz, respectively.

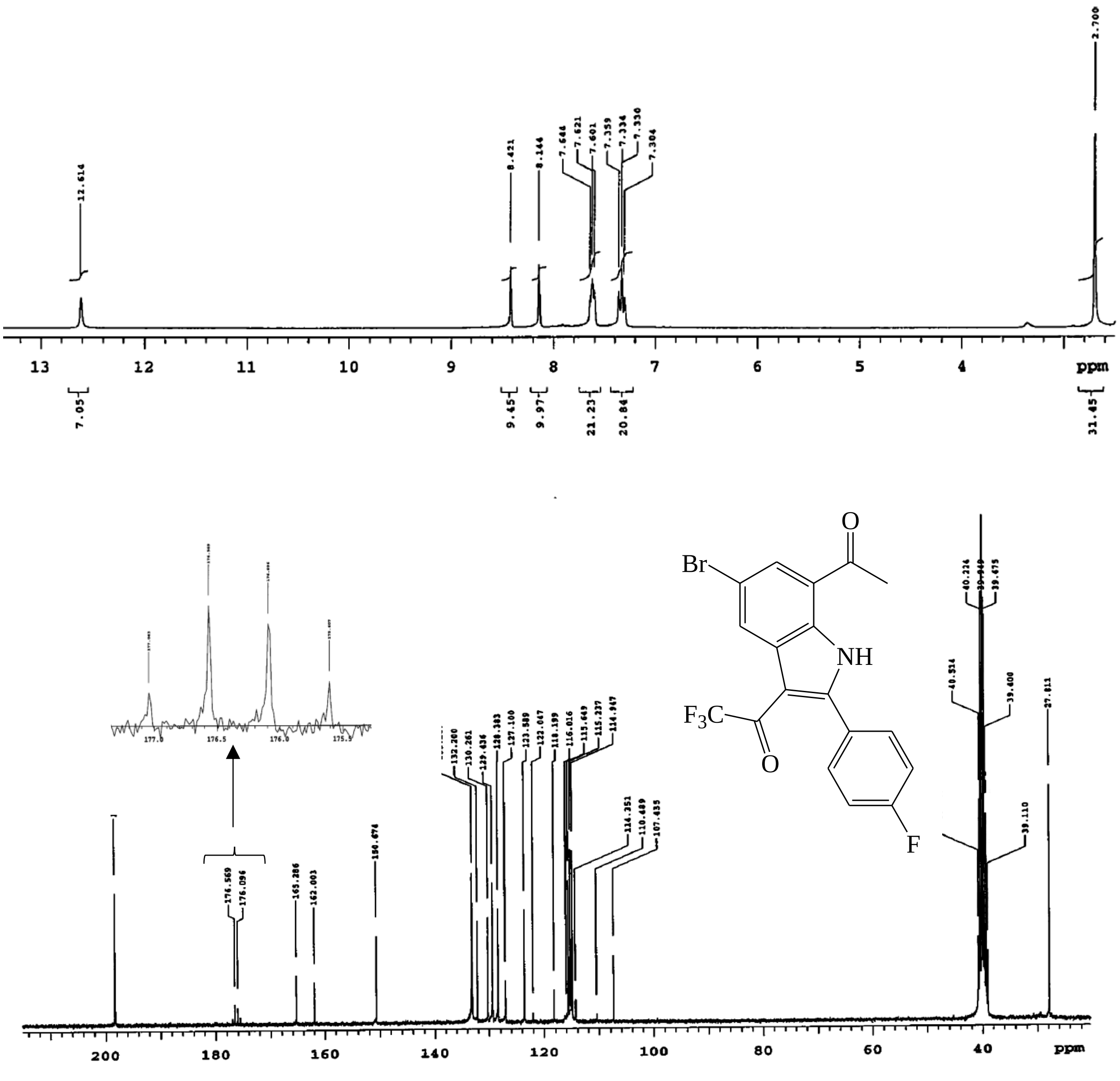

Figure S1.14: ^{1}H- and ^{13}C-NMR spectra of **5b** in DMSO-d_6 at 300 MHz and 75 MHz, respectively.

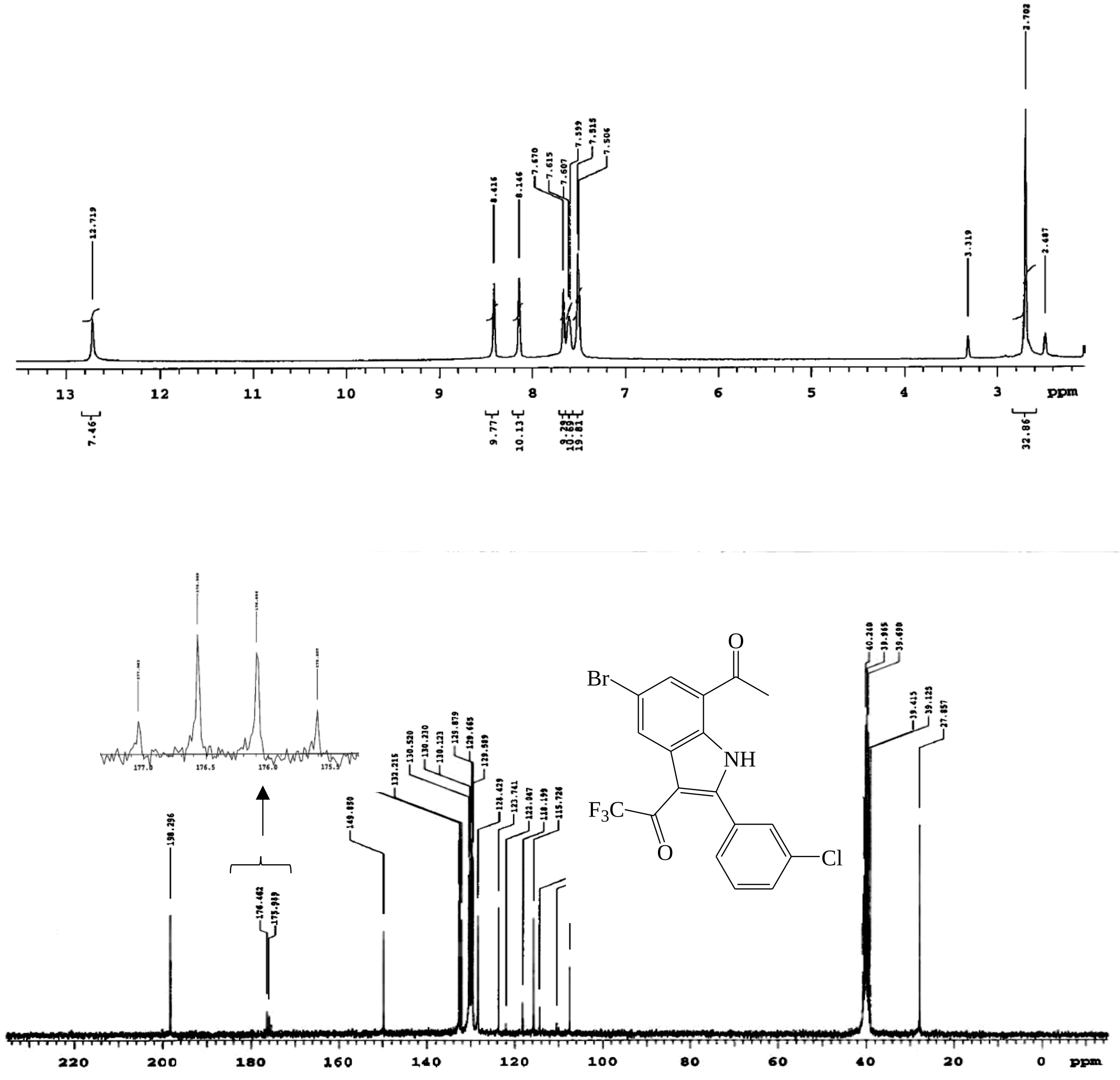

Figure S1.15: ^{1}H- and ^{13}C-NMR spectra of **5c** in DMSO-d_6 at 300 MHz and 75 MHz, respectively.

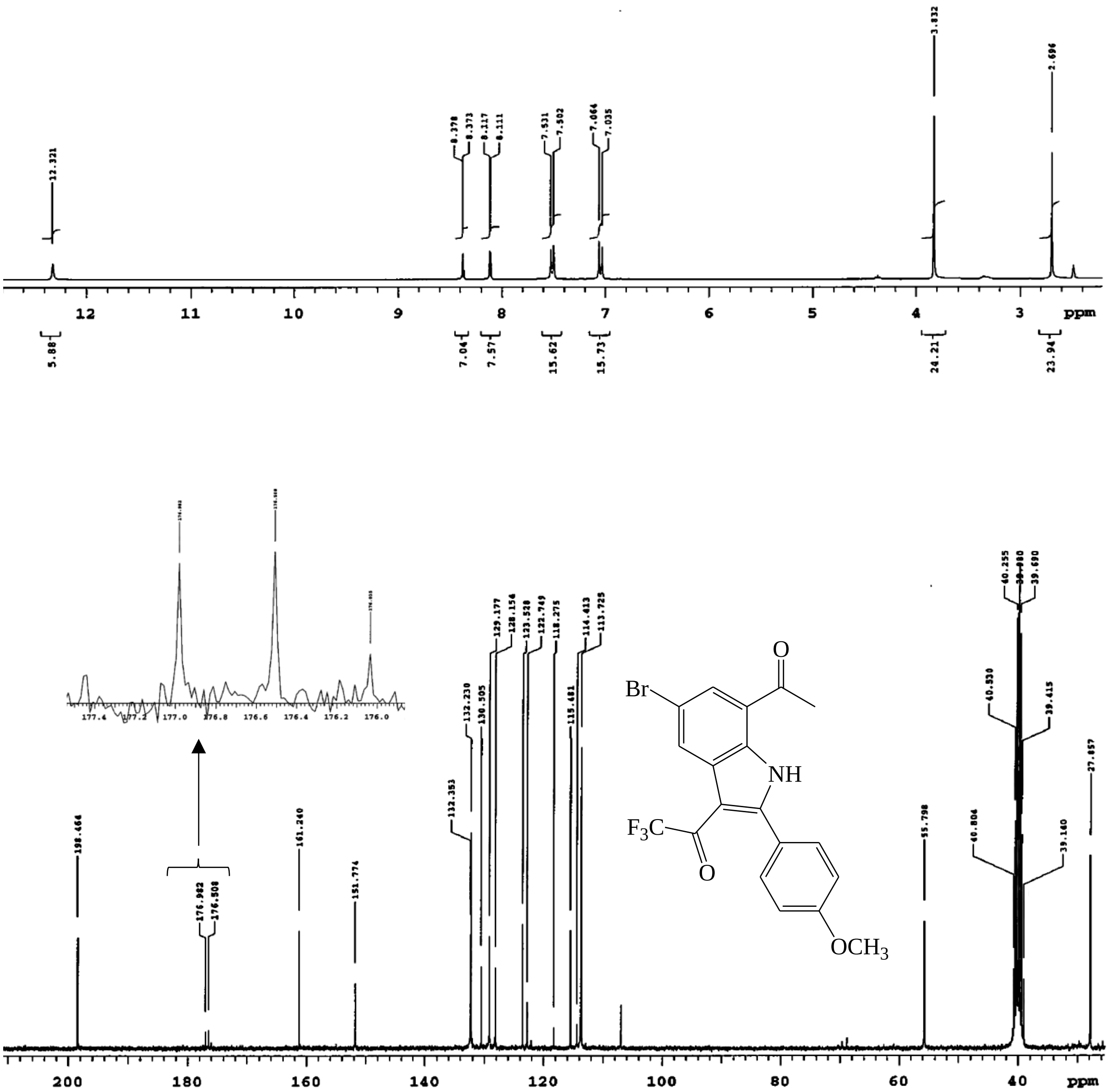

Figure S1.16: ^{1}H- and ^{13}C-NMR spectra of **5d** in DMSO-d_6 at 300 MHz and 75 MHz, respectively.

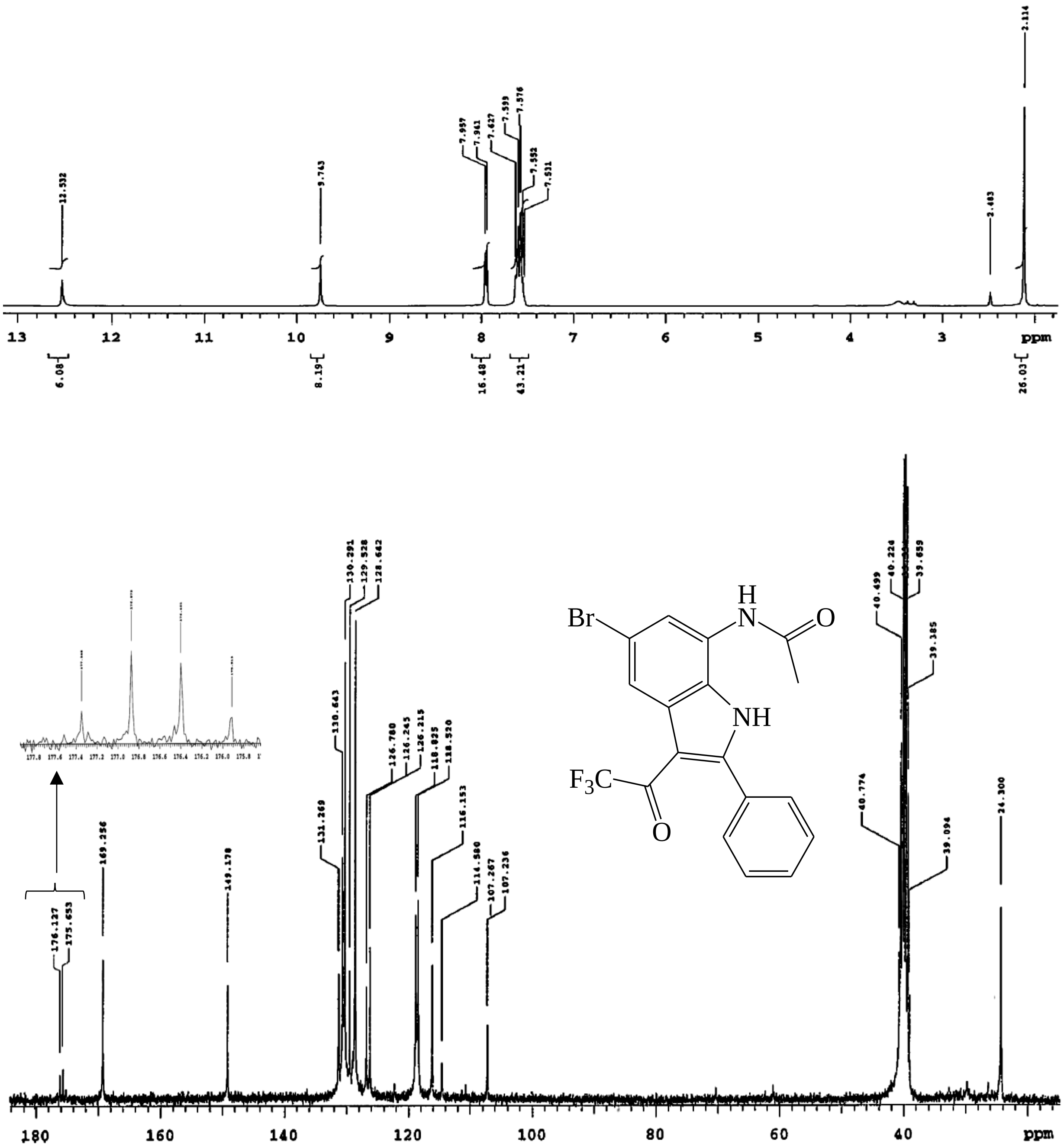

Figure S1.17: ^{1}H- and ^{13}C-NMR spectra of **5e** in DMSO-d_6 at 300 MHz and 75 MHz, respectively.

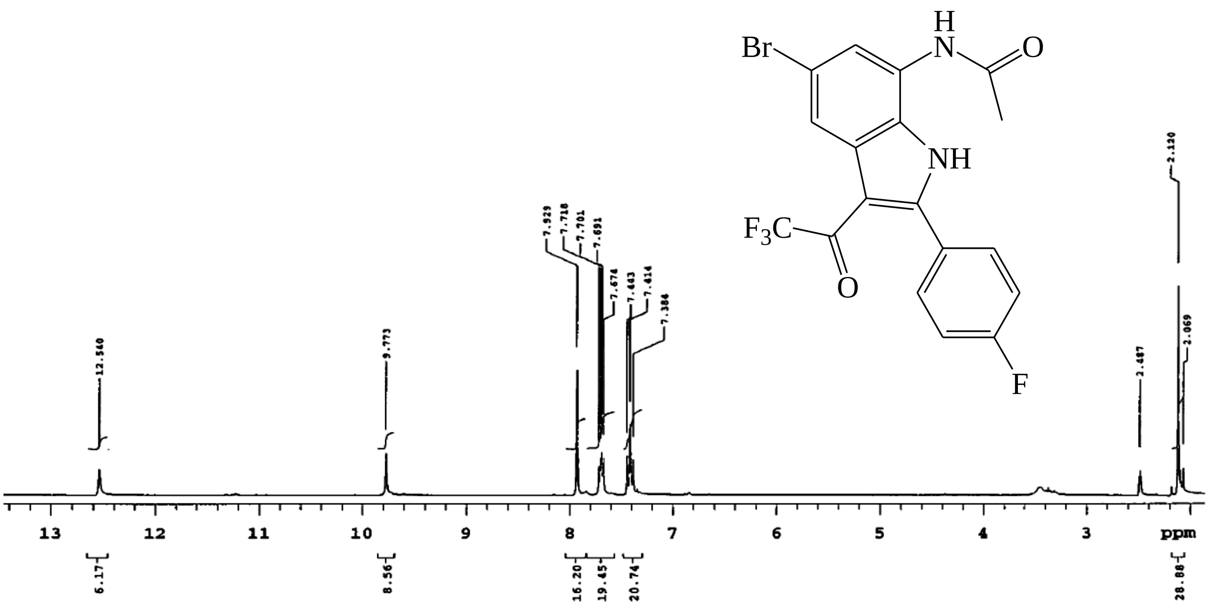

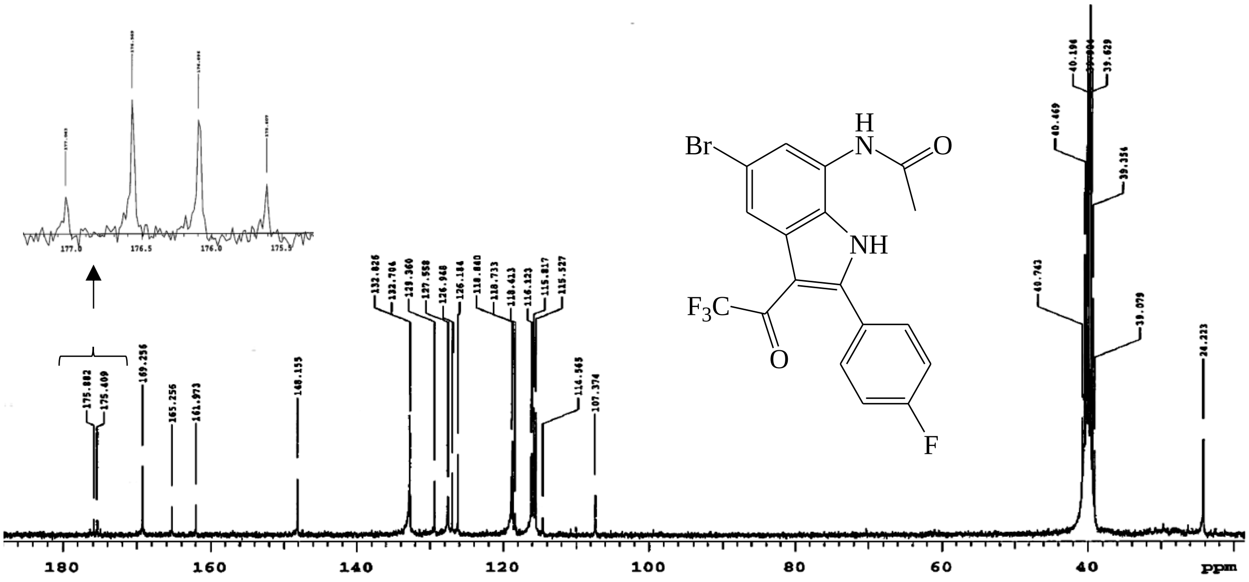

Figure S1.18: ^{1}H- and ^{13}C-NMR spectra of **5f** in DMSO-d_6 at 300 MHz and 75 MHz, respectively.

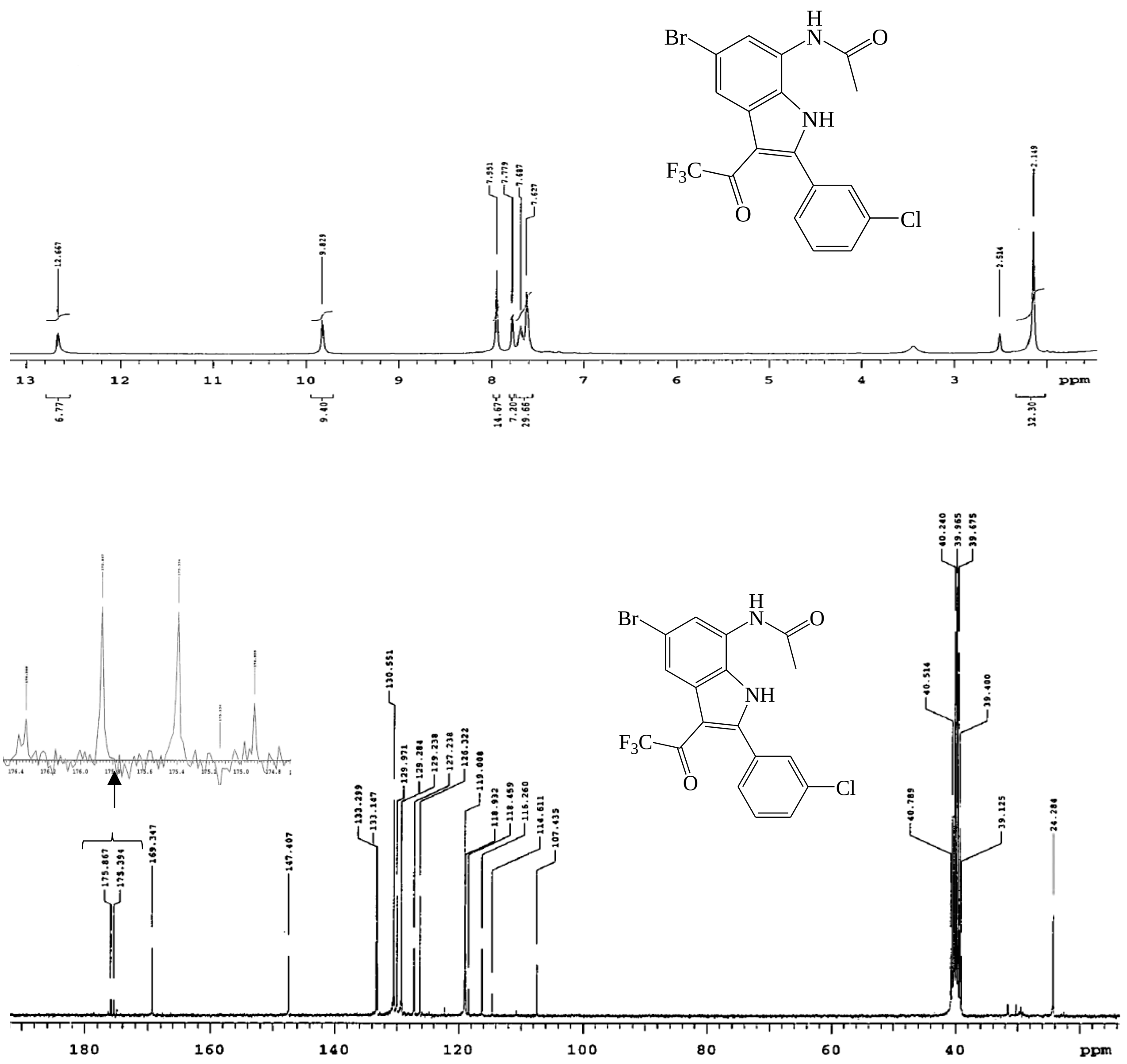

Figure S1.19: ^{1}H- and ^{13}C-NMR spectra of **5g** in DMSO-d_6 at 300 MHz and 75 MHz, respectively.

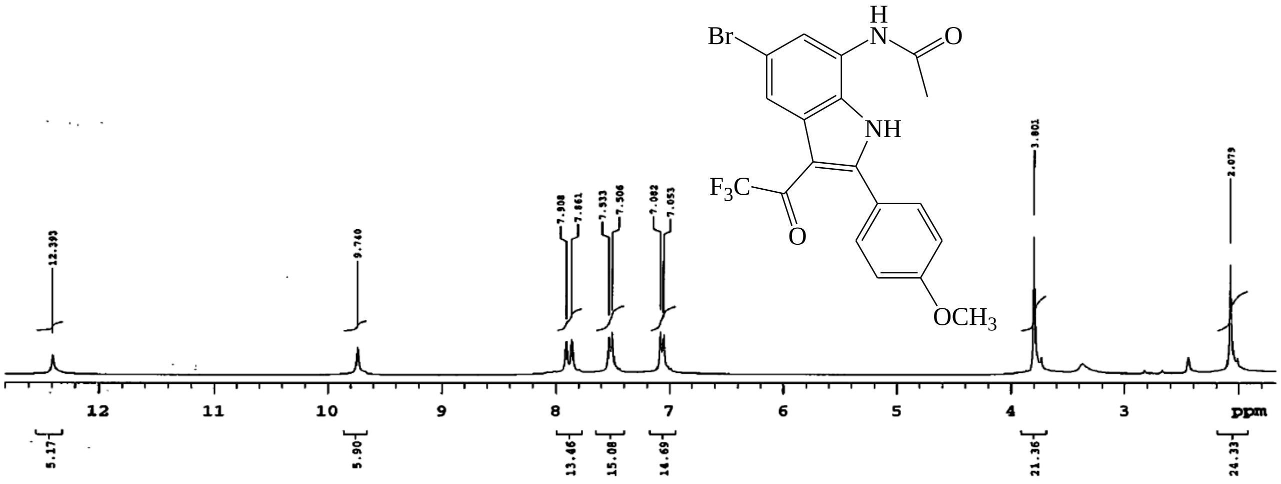

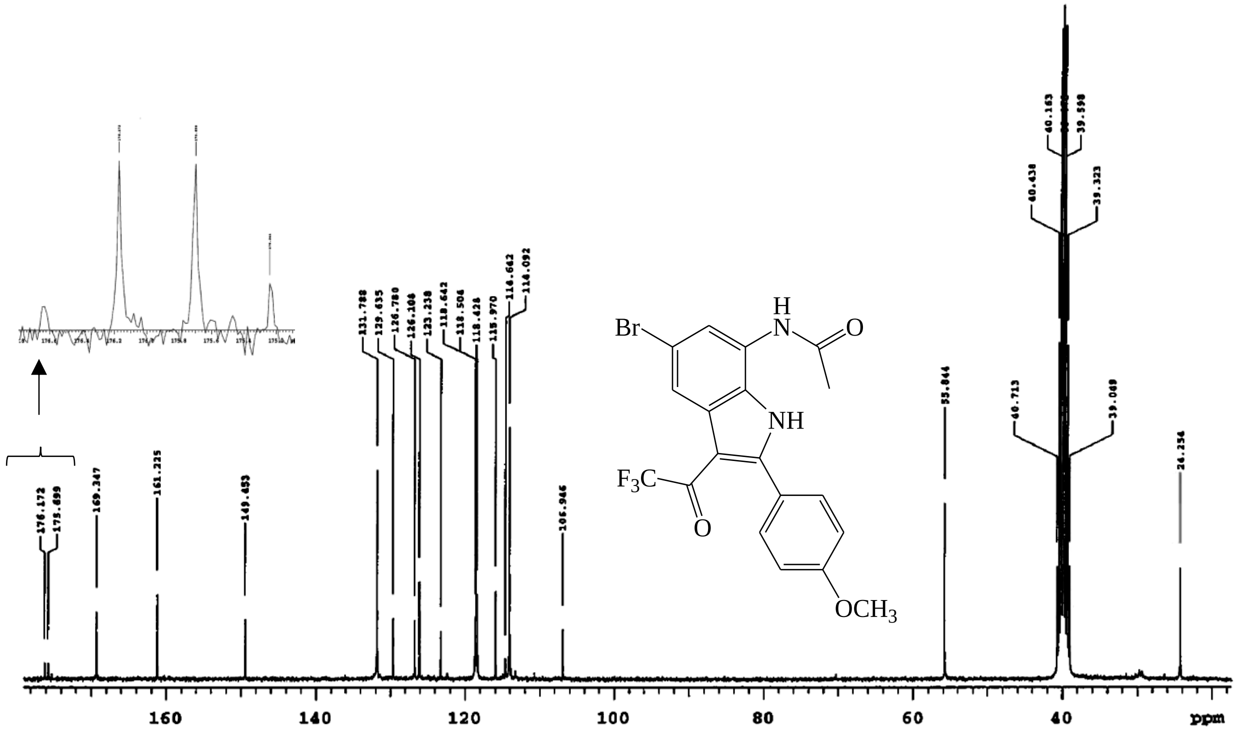

Figure S1.20: ^{1}H- and ^{13}C-NMR spectra of **5h** in DMSO-d_6 at 300 MHz and 75 MHz, respectively.

Figure S2: Cytotoxicity of compounds **2a–d**, **4a–d** and **5a–h** against A549 and HeLa cells

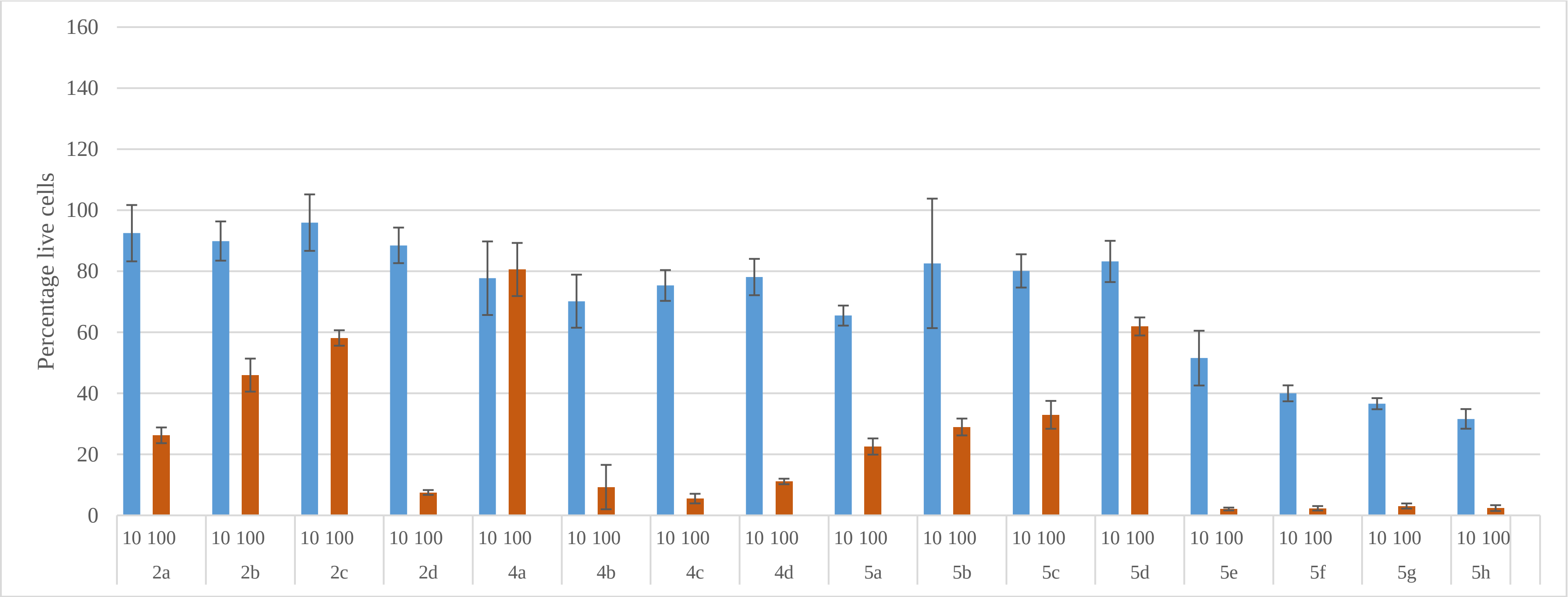

Figure S2.1: Cytotoxicity of **2a–d**, **4a–d** and **5a–h** at 10 µM (blue bar) and 100 µM (orange bar) against A549 cells**.** The results are indicated as percentage live cells compared to an untreated control. Error bars denote standard deviation of one experiment performed in quadruplicate.

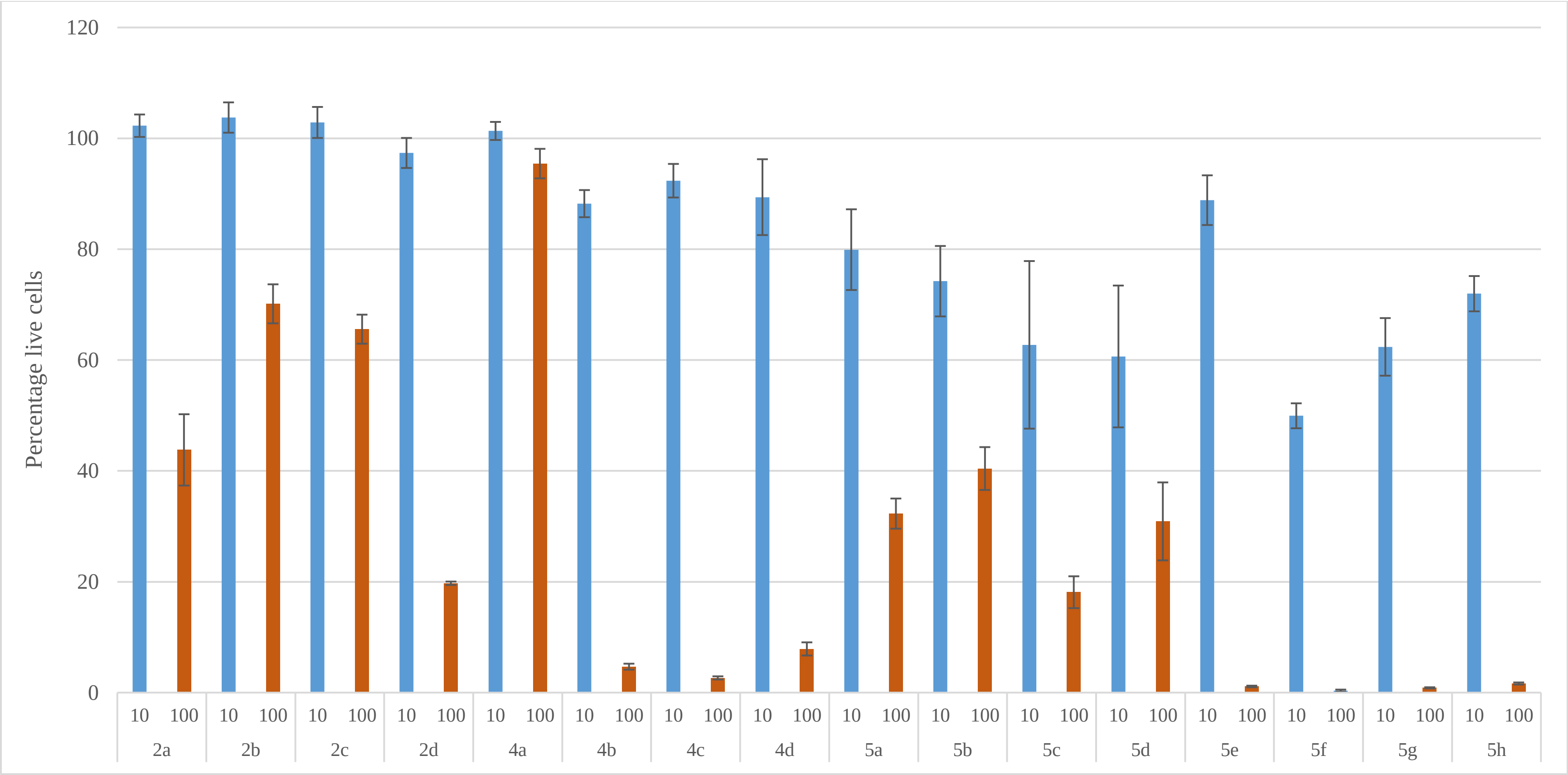

Figure S2.2: Cytotoxicity of **2a–d**, **4a–d** and **5a–h** at 10 µM (blue bar) and 100 µM (orange bar) against HeLa cervical cancer cells. The results are indicated as percentage live cells compared to an untreated control. Error bars denote standard deviation of one experiment performed in quadruplicate.

Figure S3: Dose response curves for **5e–h** against the A549 and HeLa cells

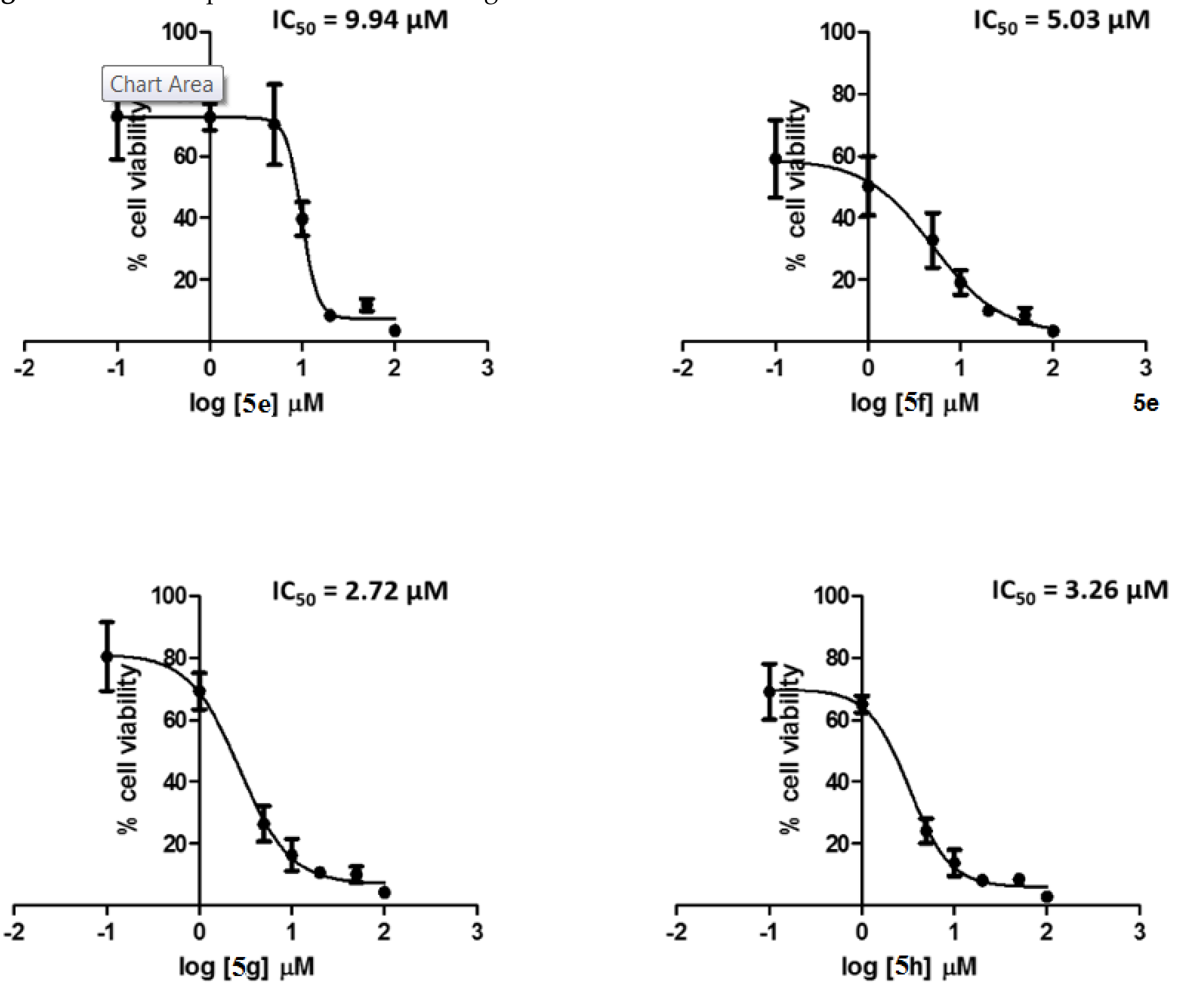

Figure S3.1: Dose response curves for compounds **5e–h** against A549 lung cancer cells. Data represents the mean ± SD for six replicate wells at each of the indicated concentrations.

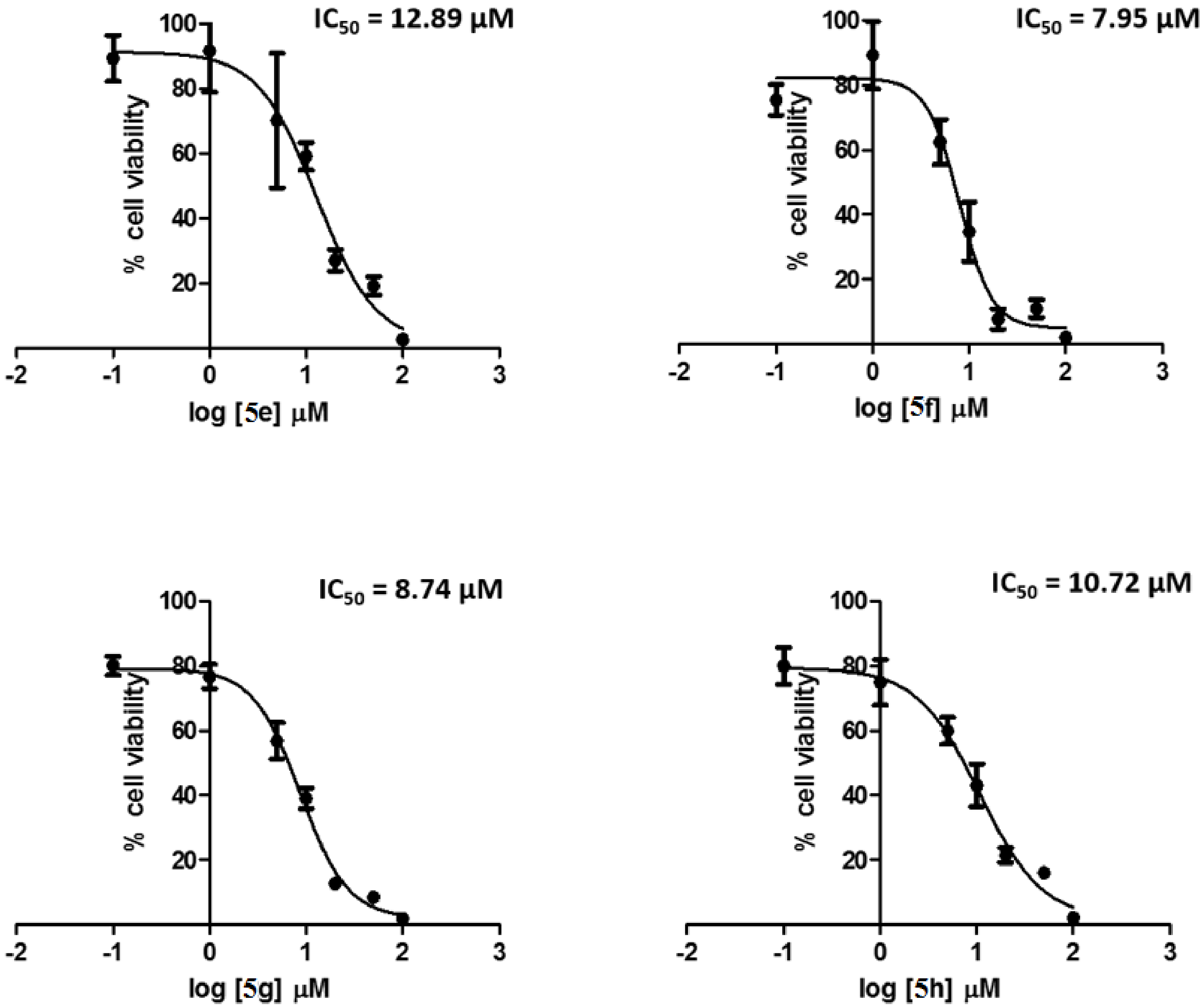

Figure 3.2: Dose response curves for **5e–h** against HeLa cells. Data represents the mean ± SD for six replicate wells at each of the indicated concentrations.

Figure S4: Spread sheet for statistical analysis which contains p values for each test

Copy of processed
results_screening of c

Copy of processed
results_screening of c

Figure S5: Excel spreadsheets for raw data and SD values for compounds **5e–h** as well as GraphPad Prism files for IC_{50} determination

RE SD and raw
data.msg
